# Race, Ethnicity, and Gender Differences in Patient Reported Well-Being and Cognitive Functioning Within 3 Months of Symptomatic Illness During COVID-19 Pandemic

**DOI:** 10.1007/s40615-024-02124-8

**Published:** 2024-08-22

**Authors:** Mandy J. Hill, Ryan M. Huebinger, Imtiaz Ebna Mannan, Huihui Yu, Lauren E. Wisk, Kelli N. O’Laughlin, Nicole L. Gentile, Kari A. Stephens, Michael Gottlieb, Robert A. Weinstein, Katherine Koo, Michelle Santangelo, Sharon Saydah, Erica S. Spatz, Zhenqiu Lin, Kevin Schaeffer, Efrat Kean, Juan Carlos C. Montoy, Robert M. Rodriguez, Ahamed H. Idris, Samuel McDonald, Joann G. Elmore, Arjun Venkatesh

**Affiliations:** 1 Department of Emergency Medicine, McGovern Medical School, UTHealth Houston, 6431 Fannin JJL 475G, Houston, TX 77030 USA; 2 Center for Outcomes Research and Evaluation, Section of Cardiovascular Medicine, Yale School of Medicine, New Haven, CT USA; 3 Division of General Internal Medicine and Health Services Research, David Geffen School of Medicine at UCLA, Los Angeles, CA USA; 4 Departments of Emergency Medicine and Global Health, University of Washington, Seattle, WA USA; 5 Department of Family Medicine, University of Washington, Seattle, WA USA; 6 Department of Laboratory Medicine and Pathology, University of Washington, Seattle, WA USA; 7 Post-COVID Rehabilitation and Recovery Clinic, University of Washington, Seattle, WA USA; 8 Biomedical Informatics & Medical Education, University of Washington, Seattle, WA USA; 9 Department of Emergency Medicine, Rush University Medical Center, Chicago, IL USA; 10 Cook County Hospital, The CORE Center, Rush University Medical Center, Chicago, IL USA; 11 Centers for Disease Control and Prevention, National Center for Immunizations and Respiratory Diseases, Atlanta, GA USA; 12 Section of Cardiovascular Medicine, Yale School of Medicine, New Haven, CT USA; 13 Department of Epidemiology, Yale School of Public Health, New Haven, CT USA; 14 Yale Center for Outcomes Research and Evaluation, New Haven, CT USA; 15 Department of Emergency Medicine, Thomas Jefferson University, Philadelphia, PA USA; 16 Center for Connected Care, Thomas Jefferson University, Philadelphia, PA USA; 17 Department of Emergency Medicine, University of California, San Francisco, San Francisco, CA USA; 18 Department of Emergency Medicine, University of Texas Southwestern Medical Center, Dallas, TX USA; 19 Clinical Informatics Center, University of Texas Southwestern Medical Center, Dallas, TX USA; 20 Department of Emergency Medicine, Yale School of Medicine, New Haven, CT USA

**Keywords:** COVID-19, Long COVID, SARS-CoV-2, Coronavirus, Registry, PROMIS outcomes

## Abstract

**Background:**

Differences in acute COVID-19 associated morbidity based on race, ethnicity, and gender have been well described; however, less is known about differences in subsequent longer term health-related quality of life and well-being.

**Methods:**

This prospective cohort study included symptomatic adults tested for SARS-CoV-2 who completed baseline and 3-month follow-up surveys. Using the PROMIS-29 tool, a validated measure of health and well-being, we compared outcomes at 3 months and change in outcomes from baseline to 3 months among groups with different races, ethnicities, and/or sexes.

**Results:**

Among 6044 participants, 4113 (3202 COVID +) were included. Among COVID + participants, compared to non-Hispanic White participants, Black participants had better PROMIS *T*-scores for cognitive function (3.6 [1.1, 6.2]) and fatigue (− 4.3 [− 6.6, − 2.0]) at 3 months and experienced more improvement in fatigue over 3 months (− 2.7 [− 4.7, − 0.8]). At 3 months, compared with males, females had worse PROMIS *T*-scores for cognitive function (− 4.1 [− 5.6, − 2.6]), physical function (− 2.1 [− 3.1, − 1.0]), social participation (− 2.8 [− 4.2, − 1.5]), anxiety (2.8 [1.5, 4.1]), fatigue (5.1 [3.7, 6.4]), and pain interference (2.0 [0.9, 3.2]). Females experienced less improvement in fatigue over 3 months (3.1 [2.0, 4.3]). Transgender/non-binary/other gender participants had worse 3-month scores in all domains except for sleep disturbance and pain interference.

**Conclusions:**

Three months after the initial COVID-19 infection, Black participants reported better cognitive function and fatigue, while females and other gender minoritized groups experienced lower well-being. Future studies are necessary to better understand how and why social constructs, specifically race, ethnicity, and gender, influence differences in COVID-19-related health outcomes.

Trials Registration

ClinicalTrials.gov Identifier: NCT04610515

**Supplementary Information:**

The online version contains supplementary material available at 10.1007/s40615-024-02124-8.

## Introduction

The SARS-CoV-2 (COVID-19) pandemic exacerbated racial-, ethnic-, and gender-based health inequities in the United States (US) [1–3]. The health impact of the COVID-19 pandemic in Black and Hispanic populations and on females has shed light on systemic factors that synergistically racially minoritized populations through social and structural constructs [4]. Between 2020 and 2021, Black people and other race and ethnic minoritized groups experienced higher COVID-19 infection rates, hospitalizations, and death when compared to White people [2–5]. Some studies have also reported gender differences, finding that compared to women with acute COVID-19, men are more likely to be hospitalized or die [5, 6]. While most studies examining racial, ethnic, and gender differences related to COVID-19 illness have focused on the acute phase, fewer have focused on the longer term outcomes [7–10].

The Innovative Support for Patients with SARS-CoV-2 Infections Registry (INSPIRE) is a multisite prospective cohort study designed to assess long-term symptoms and outcomes of participants using validated measures of health status including the National Institute of Health’s (NIH) Patient Reported Outcome Measurement Information System (PROMIS®). PROMIS can provide meaningful, patient-reported insights into patients’ experience of illness that are not captured by traditional morbidity measures [11, 12]. Wisk and colleagues (2022) assessed how long-term sequelae after an acute COVID-19 illness can impact physical, mental, and social well-being at 3 months following enrolment in the INSPIRE study [7]. Study findings highlighted the importance of including a control group of participants who experience symptoms of an acute illness, but tested negative for COVID-19 for comparison purposes. The study also did not explore differences between groups based on race and ethnic or gender identities. Although racial-, ethnic-, and gender-based differences in PROMIS related patient function scores after an acute COVID-19 illness have been observed in prior research [5], long-term PROMIS scores have not been examined and prior work has been limited to single study sites or narrow populations such as those hospitalized with an acute COVID-19 illness early in the pandemic [13, 14].

Accordingly, we sought to utilize the diverse, multi-state sample of adults who had acute symptoms suggestive of COVID-19 at time of enrollment and either tested positive (COVID +) or negative (COVID −) for SARS-CoV-2. PROMIS outcomes at baseline and over a 3-month period (baseline to 3 months) were examined based on race, ethnicity, and gender.

## Methods

### Study Design

**Fig. 1:**
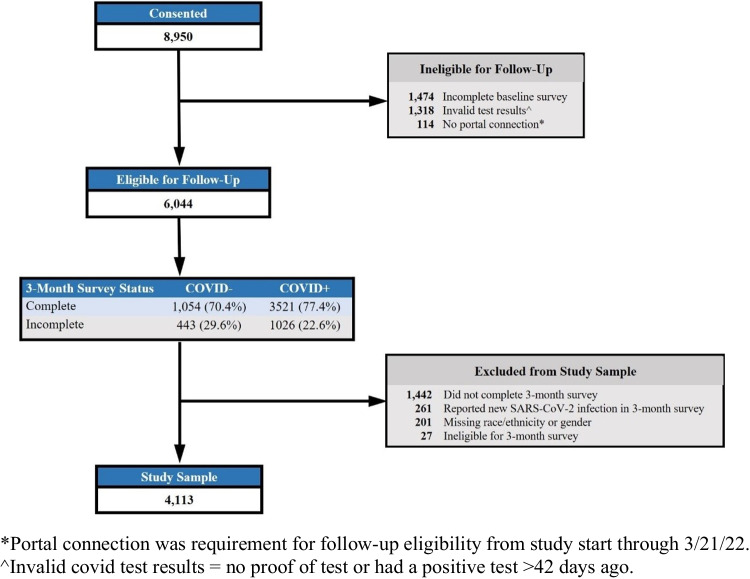
INSPIRE participant flowchart

This is a secondary analysis of data from the INSPIRE study, a multicenter, longitudinal cohort study of the sequelae of COVID-19 in the US. Participants were recruited via multiple mechanisms including emergency departments, outreach through electronic health records, and through partnerships with local and state public health departments. Inclusion criteria included age ≥ 18 years, fluency in English or Spanish, reported symptoms of COVID-19 infection (i.e., fever, shortness of breath, cough) and completion of an antigen or polymerase chain reaction (PCR) test for COVID-19 within the preceding 42 days. Exclusion criteria included inability to provide consent, being lawfully imprisoned, inability of the study team to confirm the result of the index diagnostic test for SARS-CoV-2, having a previous SARS-CoV-2 infection > 42 days before enrollment, and lacking access to an internet-connected device (e.g., smartphone, tablet, computer) for electronic survey completion. A total of 8950 adults were consented between December 7, 2020 and August 29, 2022, of which 6044 were followed prospectively with patient reported outcomes collected every 3 months for an 18-month time period (Fig. 1). Further details regarding the methods of the parent INSPIRE study were previously reported [7, 15–22].

### Study Sample

In this study, we focus on reporting results among COVID + participants to assess for differences in self-reported insights into patients’ experience with an acute illness by race and ethnicity, and gender following a COVID-19 infection. The COVID − cohort was included in the analysis to account for non-COVID-19 differences. This analysis included INSPIRE participants who completed both baseline and 3-month follow-up surveys. We categorized participants by index COVID-19 test results into COVID + and COVID − participants and primarily focused on reported results among the COVID + participants. Participants with an initial negative test were included in the analysis to account for the effects of non-COVID-19 illness in obtaining study estimates. COVID − participants who reported a positive test within 7 days of initial testing were designated as COVID + . We excluded participants who subsequently reported testing positive for COVID-19 within 3 months (*n* = 261). There were 201 participants with either missing race and ethnicity (*n* = 111) or gender (*n* = 132) or both (*n* = 42) who were also excluded. Additional inclusion and exclusion criteria are described in Fig. 1.

### Independent Variables

Participant sociodemographic characteristics included age (18–34, 35–49, 50–64, 65 + years), education (less than high school, high school, some college, 2-year degree, and 4-year degree or greater), marital status (married/living with a partner, divorced/widowed/separated, never married), health insurance status (private, public, private and public, none), annual family income in US dollars (< 35,000, 35,000–49,999, 50,000–74,999, 75,000 and more, prefer not to answer), and employment status (essential worker, non-essential worker, and unemployed). Health characteristics included tobacco use (daily or near daily, weekly, monthly, less than monthly, not at all), pre-existing conditions, hospitalization for COVID-like symptoms, baseline COVID-19 testing locations (at home testing kit, clinic including urgent care, emergency department or hospital, test/drive up testing site, and other), and COVID-19 vaccination status.

For the analysis of race/ethnicity, the exposure variable of race and ethnicity included four non-Hispanic groups (White, Black, Asian, Other) and one Hispanic/Latino (Hispanic) group. The exposure variable of gender had three categories: female, male, and transgender/non-binary/other.

### Outcomes

Our primary outcomes were the PROMIS *T*-scores and pain intensity score (range from 0, no pain to 10, worst imaginable pain). We calculated the raw scores at 3 months based on responses to PROMIS-29v2.1 questions of physical function, social participation, anxiety, depression, fatigue, sleep disturbance, pain interference, and responses to PROMIS cognitive short form 8a. PROMIS *T*-scores were obtained through the raw-score-to-*T*-score crosswalk [23]. The PROMIS T-scores have a mean of 50 and a standard deviation of 10 among the general population [23–26]. For physical function, cognitive function, and social participation, higher scores indicate better function, while for the other domains and the pain intensity scores, higher scores indicate worse function.

### Statistical Analysis

We conducted *χ*^2^ tests to compare the sociodemographic and clinical characteristics among racial–ethnic and gender groups. We used generalized linear models to estimate the difference in PROMIS *T*-scores at 3 months among race and ethnicity groups or among gender groups. We included sociodemographic characteristics, index COVID-19 test results, baseline COVID-19 testing location, health characteristics, and the interactions between exposure variables and index COVID-19 test results for risk adjustment. We further examined the racial-ethnic and gender difference in change scores over the 3 months in each COVID group by additionally adjusting for baseline PROMIS *T*-scores. We provided two examples to illustrate how adjusted marginal differences were calculated in Appendices 1a and 1b. A *p*-value of < 0.05 was considered statistically significant. A score difference of 2 or greater for PROMIS *T*-scores or of 1 or greater for pain intensity scores was considered clinically significant [7]. Given the exploratory nature of this analysis, no adjustments were made for multiple comparisons. The statistical analyses were conducted using SAS v9.4 (SAS Institute Inc) and R v4.2.2 (R Foundation for Statistical Computing).

## Results

Among 6044 participants eligible for the 3-month survey, 4575 (75.7%) completed the survey, of whom 4113 were included in the analysis after exclusion (Fig. 1). The 3-month survey completion rates ranked from high to low were Asian (78.8%), White (78.0%), Other race (75.6%), Hispanic/Latino (69.6%), and Black (57.9%) by race/ethnicity, and were transgender/non-binary/other gender (76.0%), female (74.8%), and male (74.2%) by gender.

### Observed Baseline Characteristics by Exposure Groups

For baseline characteristics, all characteristics differed by race and ethnicity groups for COVID + participants except for a few pre-existing clinical conditions. For COVID − participants, all characteristics were different between race and ethnicity groups except gender, tobacco use, a few pre-existing clinical conditions, vaccination status, and hospitalization for acute illness (Table 1). Comparing baseline characteristics across gender groups for COVID + patients, race-ethnicity, age, marital status, income, employment, tobacco use, a few pre-existing conditions, and testing location differed. For COVID − patients, age, marital status, income, employment, tobacco use, a few pre-existing conditions, and testing location differed amongst gender groups (Table 2).

**Table 1:** Characteristics of participants by race/ethnicity and index COVID-19 test results

	*COVID* +						*COVID-*					
	*Non-Hispanic*				*Hispanic/* *Latino* *(N* = *438)*	*P-value*	*Non-Hispanic*				*Hispanic/* *Latino* *(N* = *152)*	*P-value*
	*White* *(N* = *1953)*	*Black* *(N* = *213)*	*Asian* *(N* = *409)*	*Other* *(N* = *1 89)*			*White* *(N* = *470)*	*Black* *(N* = *109)*	*Asian* *(N* = *140)*	*Other* *(N* = *40)*		
	*n (%)*	*n (%)*	*n(%)*	*n (%)*	*n (%)*		*n (%)*	*n (%)*	*n (%)*	*n (%)*	*n (%)*	
Gender						**0.020**						**0.57**
Female	1281 (65.6)	160 (75.1)	261 (63.8)	131 (69.3)	302 (68.9)		325 (69.1)	81 (74.3)	96 (68.6)	31 (77.5)	110 (72.4)	
Male	644 (33.0)	53 (24.9)	147 (35.9)	55 (29.1)	129 (29.5)		130 (27.7)	28 (25.7)	41 (29.3)	9 (22.5)	39 (25.7)	
Transgender/non-binary Other	28 (1.4)	0 (0.0)	1 (0.2)	3 (1.6)	7 (1.6)		15 (3.2)	0 (0.0)	3 (2.1)	0 (0.0)	3 (2.0)	
Age						** < 0.001**						** < 0.001**
18–34	704 (36.0)	80 (37.6)	235 (57.5)	84 (44.4)	235 (53.7)		186 (39.6)	38 (34.9)	95 (67.9)	27 (67.5)	73 (48.0)	
35–49	649 (33.2)	73 (34.3)	124 (30.3)	69 (36.5)	135 (30.8)		125 (26.6)	32 (29.4)	32 (22.9)	8 (20.0)	54 (35.5)	
50–64	399 (20.4)	50 (23.5)	37 (9.0)	32 (16.9)	55 (12.6)		95 (20.2)	30 (27.5)	10 (7.1)	5 (12.5)	22 (14.5)	
65 +	201 (10.3)	10 (4.7)	13 (3.2)	4 (2.1)	13 (3.0)		64 (13.6)	9 (8.3)	3 (2.1)	0 (0.0)	3 (2.0)	
Education						** < 0.001**						** < 0.001**
Less than high school	9 (0.5)	6 (2.8)	2 (0.5)	1 (0.5)	8 (1.9)		4 (0.9)	5 (4.7)	0 (0.0)	0 (0.0)	6 (4.0)	
High school graduate	75 (3.9)	39 (18.5)	17 (4.2)	10 (5.4)	44 (10.2)		36 (7.8)	18 (17.0)	15 (10.9)	5 (12.5)	20 (13.4)	
Some college	207 (10.7)	51 (24.2)	30 (7.4)	34 (18.5)	92 (21.3)		66 (14.3)	26 (24.5)	29 (21.2)	11 (27.5)	32 (21.5)	
2-year degree	109 (5.7)	24 (11.4)	12 (3.0)	14 (7.6)	44 (10.2)		37 (8.0)	9 (8.5)	8 (5.8)	2 (5.0)	14 (9.4)	
4-year degree	698 (36.2)	47 (22.3)	157 (38.7)	72 (39.1)	124 (28.8)		119 (25.8)	24 (22.6)	35 (25.5)	8 (20.0)	40 (26.8)	
More than 4 years	831 (43.1)	44 (20.9)	188 (46.3)	53 (28.8)	119 (27.6)		199 (43.2)	24 (22.6)	50 (36.5)	14 (35.0)	37 (24.8)	
Marital status						** < 0.001**						** < 0.001**
Married/living with a partner	1185 (60.7)	77 (36.2)	202 (49.4)	88 (46.8)	237 (54.1)		245 (52.4)	30 (27.5)	43 (30.7)	16 (40.0)	64 (42.1)	
Divorced/widowed/separated	194 (9.9)	28 (13.1)	16 (3.9)	23 (12.2)	29 (6.6)		61 (13.0)	30 (27.5)	6 (4.3)	6 (15.0)	13 (8.6)	
Never married	573 (29.4)	108 (50.7)	191 (46.7)	77 (41.0)	172 (39.3)		162 (34.6)	49 (45.0)	91 (65.0)	18 (45.0)	75 (49.3)	
Family income						** < 0.001**						** < 0.001**
< 10,000	55 (2.8)	28 (13.1)	24 (5.9)	14 (7.4)	29 (6.6)		25 (5.3)	18 (16.5)	18 (12.9)	3 (7.5)	17 (11.2)	
10,000–34,999	139 (7.1)	51 (23.9)	24 (5.9)	21 (11.1)	84 (19.2)		50 (10.6)	30 (27.5)	11 (7.9)	8 (20.0)	30 (19.7)	
35,000–49,999	147 (7.5)	38 (17.8)	33 (8.1)	21 (11.1)	67 (15.3)		55 (11.7)	17 (15.6)	16 (11.4)	5 (12.5)	30 (19.7)	
50,000–74,999	251 (12.9)	34 (16.0)	50 (12.2)	20 (10.6)	66 (15.1)		63 (13.4)	21 (19.3)	21 (15.0)	5 (12.5)	17 (11.2)	
75,000 +	1265 (64.8)	52 (24.4)	236 (57.7)	96 (50.8)	156 (35.6)		247 (52.6)	14 (12.8)	52 (37.1)	10 (25.0)	45 (29.6)	
Prefer not to answer	96 (4.9)	10 (4.7)	42 (10.3)	17 (9.0)	36 (8.2)		30 (6.4)	9 (8.3)	22 (15.7)	9 (22.5)	13 (8.6)	
Health insurance						** < 0.001**						** < 0.001**
Private only	1536 (78.6)	117 (54.9)	342 (83.6)	142 (75.1)	307 (70.1)		324 (68.9)	45 (41.3)	108 (77.1)	31 (77.5)	102 (67.1)	
Public only	287 (14.7)	79 (37.1)	54 (13.2)	37 (19.6)	86 (19.6)		111 (23.6)	57 (52.3)	26 (18.6)	7 (17.5)	38 (25.0)	
Private and public	85 (4.4)	7 (3.3)	6 (1.5)	4 (2.1)	9 (2.1)		28 (6.0)	1 (0.9)	2 (1.4)	2 (5.0)	2 (1.3)	
None	45 (2.3)	10 (4.7)	7 (1.7)	6 (3.2)	36 (8.2)		7 (1.5)	6 (5.5)	4 (2.9)	0 (0.0)	10 (6.6)	
Employment						**0.041**						**0.040**
Employed, essential	802 (41.1)	103 (48.4)	170 (41.6)	76 (40.2)	207 (47.4)		178 (37.9)	51 (46.8)	42 (30.0)	17 (42.5)	62 (40.8)	
Employed, non-essential	833 (42.7)	69 (32.4)	171 (41.8)	79 (41.8)	153 (35.0)		179 (38.1)	33 (30.3)	49 (35.0)	9 (22.5)	56 (36.8)	
Not employed	315 (16.2)	41 (19.2)	68 (16.6)	34 (18.0)	77 (17.6)		113 (24.0)	25 (22.9)	49 (35.0)	14 (35.0)	34 (22.4)	
Tobacco use						**0.034**						**0.05**
Daily or near daily	101 (5.2)	16 (7.5)	13 (3.2)	11 (5.8)	17 (3.9)		40 (8.5)	9 (8.3)	4 (2.9)	2 (5.0)	12 (7.9)	
Weekly	27 (1.4)	7 (3.3)	4 (1.0)	3 (1.6)	6 (1.4)		7 (1.5)	5 (4.6)	4 (2.9)	2 (5.0)	4 (2.6)	
Monthly	27 (1.4)	1 (0.5)	6 (1.5)	6 (3.2)	6 (1.4)		3 (0.6)	2 (1.8)	6 (4.3)	2 (5.0)	3 (2.0)	
Less than monthly	90 (4.6)	15 (7.0)	19 (4.6)	16 (8.5)	28 (6.4)		19 (4.0)	4 (3.7)	6 (4.3)	1 (2.5)	12 (7.9)	
Not at all	1707 (87.4)	174 (81.7)	367 (89.7)	153 (81.0)	381 (87.0)		401 (85.3)	89 (81.7)	120 (85.7)	33 (82.5)	121 (79.6)	
Pre-existing conditions												
Asthma	198 (10.3)	40 (19.8)	40 (9.9)	40 (21.6)	48 (11.2)	< 0.001	74 (15.8)	21 (19.8)	13 (9.4)	10 (25.6)	23 (15.2)	0.07
Hypertension	249 (12.9)	50 (24.8)	30 (7.4)	27 (14.6)	45 (10.5)	< 0.001	82 (17.6)	28 (26.4)	9 (6.5)	6 (15.4)	15 (9.9)	< 0.001
Diabetes	74 (3.8)	19 (9.4)	13 (3.2)	14 (7.6)	19 (4.4)	< 0.001	29 (6.2)	17 (16.0)	4 (2.9)	2 (5.1)	9 (6.0)	0.001
Obesity	495 (25.7)	79 (39.1)	52 (12.8)	55 (29.7)	136 (31.7)	< 0.001	143 (30.6)	34 (32.1)	20 (14.4)	12 (30.8)	51 (33.8)	0.002
Emphysema/COPD	13 (0.7)	5 (2.5)	0 (0.0)	0 (0.0)	1 (0.2)	0.002	10 (2.1)	5 (4.7)	0 (0.0)	0 (0.0)	2 (1.3)	0.08
Heart conditions	42 (2.2)	8 (4.0)	4 (1.0)	4 (2.2)	4 (0.9)	0.06	22 (4.7)	6 (5.7)	2 (1.4)	0 (0.0)	4 (2.6)	0.18
Smoking/tobacco consumption	75 (3.9)	19 (9.4)	9 (2.2)	13 (7.0)	10 (2.3)	< 0.001	32 (6.9)	6 (5.7)	5 (3.6)	2 (5.1)	9 (6.0)	0.72
Kidney disease	19 (1.0)	6 (3.0)	4 (1.0)	2 (1.1)	2 (0.5)	0.07	10 (2.1)	4 (3.8)	2 (1.4)	1 (2.6)	1 (0.7)	0.49
Liver disease	16 (0.8)	3 (1.5)	0 (0.0)	0 (0.0)	1 (0.2)	0.08	8 (1.7)	3 (2.8)	1 (0.7)	0 (0.0)	3 (2.0)	0.66
None	421 (21.8)	20 (9.9)	82 (20.2)	34 (18.4)	58 (13.5)	< 0.001	58 (12.4)	10 (9.4)	29 (20.9)	13 (33.3)	18 (11.9)	< 0.001
I do not know	422 (21.9)	25 (12.4)	145 (35.7)	33 (17.8)	111 (25.9)	< 0.001	75 (16.1)	18 (17.0)	44 (31.7)	6 (15.4)	35 (23.2)	< 0.001
Prefer not to answer	86 (4.5)	10 (5.0)	24 (5.9)	12 (6.5)	31 (7.2)	0.15	25 (5.4)	7 (6.6)	18 (12.9)	1 (2.6)	12 (7.9)	0.027
COVID-19 vaccination status^a^						** < 0.001**						**0.11**
Yes	1195 (76.2)	95 (53.7)	265 (87.7)	108 (77.1)	255 (73.1)		319 (81.0)	67 (83.8)	92 (86.0)	23 (71.9)	91 (74.0)	
No	374 (23.8)	82 (46.3)	37 (12.3)	32 (22.9)	94 (26.9)		75 (19.0)	13 (16.3)	15 (14.0)	9 (28.1)	32 (26.0)	
Baseline testing location						** < 0.001**						** < 0.001**
At home testing kit	326 (16.8)	16 (7.5)	77 (18.8)	28 (14.8)	50 (11.4)		55 (11.7)	9 (8.3)	23 (16.4)	5 (12.5)	11 (7.2)	
Clinic including urgent care	260 (13.4)	29 (13.7)	24 (5.9)	20 (10.6)	60 (13.7)		101 (21.5)	13 (11.9)	16 (11.4)	8 (20.0)	29 (19.1)	
Emergency department	41 (2.1)	27 (12.7)	5 (1.2)	10 (5.3)	9 (2.1)		25 (5.3)	25 (22.9)	7 (5.0)	2 (5.0)	9 (5.9)	
Hospital	119 (6.1)	44 (20.8)	27 (6.6)	15 (7.9)	44 (10.1)		46 (9.8)	18 (16.5)	8 (5.7)	5 (12.5)	15 (9.9)	
Other	119 (6.1)	10 (4.7)	25 (6.1)	12 (6.3)	42 (9.6)		50 (10.7)	7 (6.4)	36 (25.7)	10 (25.0)	26 (17.1)	
Tent/drive up testing site	1081 (55.5)	86 (40.6)	251 (61.4)	104 (55.0)	232 (53.1)		192 (40.9)	37 (33.9)	50 (35.7)	10 (25.0)	62 (40.8)	
Hospitalized at time of acute COVID-like symptoms^b^						** < 0.001**						**0.73**
ICU stay	14 (0.7)	14 (6.9)	2 (0.5)	5 (2.7)	7 (1.6)		1 (0.2)	1 (0.9)	0 (0.0)	0 (0.0)	0 (0.0)	
Hospitalized (non-ICU)	44 (2.3)	22 (10.9)	12 (3.0)	4 (2.2)	20 (4.7)		5 (1.1)	1 (0.9)	0 (0.0)	0 (0.0)	2 (1.3)	
Not hospitalized	1874 (97.0)	166 (82.2)	392 (96.6)	177 (95.2)	401 (93.7)		462 (98.7)	107 (98.2)	140 (100.0)	39 (100.0)	150 (98.7)	

^a^SARS-CoV-2 vaccination status indicates participants with at least one dose prior to the index COVID-19 test; Vaccination initiation information was obtained from linked electronic health record data and patient survey responses

^b^Hospitalized for COVID-like symptoms questions not asked on baseline; these questions are asked on each quarterly Follow-up Survey beginning 4-14-2021; the section shown here includes combined data from the 3mo f/u surveys

Pre-existing conditions questions were only asked on 3-Month Follow-up Survey beginning 4-14-2021 and have missing data for COVID + (*n* = 51) and COVID − (*n* = 9)

Yellow-labelled cells indicate some cells in the corresponding variable have expected counts < 5 under null hypothesis; all *p*-values are calculated using chi-square test. Chi-square tests were conducted separately within each COVID groups. * ≥ *p*-value < 0.05, ** ≥ *p*-value < 0.01, *** ≥ *p*-value < 0.001

**Table 2:** Characteristics of participants by gender and index COVID-19 test results

	*COVID* +				*COVID-*			
	*Male (N* = *1028)*	*Female (N* = *2135)*	*Transgender/Non-binary Other (N* = *39)*	*P-value*	*Male (N* = *247)*	*Female (N* = *643)*	*Transgender/Non-binary Other (N* = *21)*	*P-value*
	*N(%)*	*N(%)*	*N(%)*		*N(%)*	*N(%)*	*N(%)*	
Race/ethnicity				0.020*				**0.57**
Non-Hispanic White	644 (62.6)	1281 (60.0)	28 (71.8)		130 (52.6)	325 (50.5)	15 (71.4)	
Non-Hispanic Black	53 (5.2)	160 (7.5)	0 (0.0)		28 (11.3)	81 (12.6)	0 (0.0)	
Non-Hispanic Asian	147 (14.3)	261 (12.2)	1 (2.6)		41 (16.6)	96 (14.9)	3 (14.3)	
Non-Hispanic Other	55 (5.4)	131 (6.1)	3 (7.7)		9 (3.6)	31 (4.8)	0 (0.0)	
Hispanic/Latino	129 (12.5)	302 (14.1)	7 (17.9)		39 (15.8)	110 (17.1)	3 (14.3)	
Age				** < 0.001**				** < 0.001**
18–34	370 (36.0)	941 (44.1)	27 (69.2)		97 (39.3)	304 (47.3)	18 (85.7)	
35–49	323 (31.4)	717 (33.6)	10 (25.6)		67 (27.1)	183 (28.5)	1 (4.8)	
50–64	224 (21.8)	348 (16.3)	1 (2.6)		48 (19.4)	113 (17.6)	1 (4.8)	
65 +	111 (10.8)	129 (6.0)	1 (2.6)		35 (14.2)	43 (6.7)	1 (4.8)	
Education				**0.26**				**0.62**
Less than high school	8 (0.8)	17 (0.8)	1 (2.6)		4 (1.7)	11 (1.7)	0 (0.0)	
High school graduate	72 (7.1)	110 (5.2)	3 (7.9)		30 (12.4)	60 (9.5)	4 (19.0)	
Some college	125 (12.3)	281 (13.3)	8 (21.1)		40 (16.5)	119 (18.9)	5 (23.8)	
2-year degree	57 (5.6)	144 (6.8)	2 (5.3)		15 (6.2)	52 (8.3)	3 (14.3)	
4-year degree	340 (33.5)	746 (35.4)	12 (31.6)		63 (26.0)	158 (25.1)	5 (23.8)	
More than 4 years	414 (40.7)	809 (38.4)	12 (31.6)		90 (37.2)	230 (36.5)	4 (19.0)	
Marital Status				** < 0.001**				**0.028**
Married/living with a partner	629 (61.2)	1142 (53.5)	18 (46.2)		119 (48.4)	275 (42.8)	4 (19.0)	
Divorced/widowed/separated	66 (6.4)	221 (10.4)	3 (7.7)		24 (9.8)	90 (14.0)	2 (9.5)	
Never married	332 (32.3)	771 (36.1)	18 (46.2)		103 (41.9)	277 (43.1)	15 (71.4)	
Family income				** < 0.001**				**0.040**
< 10,000	43 (4.2)	107 (5.0)	0 (0.0)		18 (7.3)	59 (9.2)	4 (19.0)	
10,000–34,999	94 (9.1)	212 (9.9)	13 (33.3)		31 (12.6)	91 (14.2)	7 (33.3)	
35,000–49,999	93 (9.0)	211 (9.9)	2 (5.1)		30 (12.1)	91 (14.2)	2 (9.5)	
50,000–74,999	98 (9.5)	313 (14.7)	10 (25.6)		35 (14.2)	88 (13.7)	4 (19.0)	
75,000 +	641 (62.4)	1153 (54.0)	11 (28.2)		115 (46.6)	249 (38.7)	4 (19.0)	
Prefer not to answer	59 (5.7)	139 (6.5)	3 (7.7)		18 (7.3)	65 (10.1)	0 (0.0)	
Health insurance				**0.39**				**0.89**
Private only	777 (75.6)	1640 (76.8)	27 (69.2)		162 (65.6)	435 (67.7)	13 (61.9)	
Public only	175 (17.0)	357 (16.7)	11 (28.2)		65 (26.3)	167 (26.0)	7 (33.3)	
Private and public	42 (4.1)	69 (3.2)	0 (0.0)		12 (4.9)	22 (3.4)	1 (4.8)	
None	34 (3.3)	69 (3.2)	1 (2.6)		8 (3.2)	19 (3.0)	0 (0.0)	
Employment				** < 0.001**				**0.030**
Employed, essential	371 (36.1)	968 (45.4)	19 (48.7)		77 (31.2)	266 (41.4)	7 (33.3)	
Employed, non-essential	499 (48.5)	792 (37.2)	14 (35.9)		98 (39.7)	217 (33.7)	11 (52.4)	
Not employed	158 (15.4)	371 (17.4)	6 (15.4)		72 (29.1)	160 (24.9)	3 (14.3)	
Tobacco use				** < 0.001**				**0.013**
Daily or near daily	67 (6.5)	86 (4.0)	5 (12.8)		24 (9.7)	41 (6.4)	2 (9.5)	
Weekly	20 (1.9)	26 (1.2)	1 (2.6)		5 (2.0)	17 (2.6)	0 (0.0)	
Monthly	20 (1.9)	26 (1.2)	0 (0.0)		4 (1.6)	10 (1.6)	2 (9.5)	
Less than monthly	65 (6.3)	94 (4.4)	9 (23.1)		18 (7.3)	22 (3.4)	2 (9.5)	
Not at all	856 (83.3)	1902 (89.1)	24 (61.5)		196 (79.4)	553 (86.0)	15 (71.4)	
Pre-existing conditions								
Asthma	92 (9.1)	269 (12.8)	5 (12.8)	0.009	28 (11.4)	110 (17.3)	3 (14.3)	0.1
Hypertension	174 (17.1)	225 (10.7)	2 (5.1)	< 0.001	44 (18.0)	94 (14.8)	2 (9.5)	0.38
Diabetes	57 (5.6)	81 (3.9)	1 (2.6)	0.07	18 (7.3)	42 (6.6)	1 (4.8)	0.86
Obesity	218 (21.5)	588 (28.0)	11 (28.2)	< 0.001	54 (22.0)	201 (31.6)	5 (23.8)	0.017
Emphysema/COPD	4 (0.4)	15 (0.7)	0 (0.0)	0.49	10 (4.1)	7 (1.1)	0 (0.0)	0.012
Heart conditions	33 (3.3)	26 (1.2)	3 (7.7)	< 0.001	21 (8.6)	12 (1.9)	1 (4.8)	< 0.001
Smoking/tobacco consumption	46 (4.5)	70 (3.3)	10 (25.6)	< 0.001	15 (6.1)	38 (6.0)	1 (4.8)	0.97
Kidney disease	12 (1.2)	21 (1.0)	0 (0.0)	0.73	13 (5.3)	5 (0.8)	0 (0.0)	< 0.001
Liver disease	7 (0.7)	13 (0.6)	0 (0.0)	0.86	6 (2.4)	9 (1.4)	0 (0.0)	0.47
None	171 (16.8)	440 (21.0)	4 (10.3)	0.008	26 (10.6)	100 (15.7)	2 (9.5)	0.12
I do not know	269 (26.5)	461 (22.0)	6 (15.4)	0.010	50 (20.4)	123 (19.3)	5 (23.8)	0.84
Prefer not to answer	55 (5.4)	106 (5.1)	2 (5.1)	0.91	18 (7.3)	43 (6.8)	2 (9.5)	0.86
Vaccination status^a^				**0.07**				**0.84**
Yes	609 (73.8)	1277 (76.2)	32 (88.9)		165 (81.3)	411 (80.0)	16 (84.2)	
No	216 (26.2)	399 (23.8)	4 (11.1)		38 (18.7)	103 (20.0)	3 (15.8)	
Testing location				**0.009**				** < 0.001**
At home testing kit	144 (14.0)	347 (16.3)	6 (15.4)		34 (13.8)	65 (10.1)	4 (19.0)	
Clinic including urgent care	102 (10.0)	288 (13.5)	3 (7.7)		36 (14.6)	123 (19.1)	8 (38.1)	
Emergency department	27 (2.6)	64 (3.0)	1 (2.6)		26 (10.6)	42 (6.5)	0 (0.0)	
Hospital	91 (8.9)	158 (7.4)	0 (0.0)		37 (15.0)	55 (8.6)	0 (0.0)	
Other	58 (5.7)	148 (7.0)	2 (5.1)		21 (8.5)	105 (16.3)	3 (14.3)	
Tent/drive up testing site	603 (58.8)	1124 (52.8)	27 (69.2)		92 (37.4)	253 (39.3)	6 (28.6)	
Hospitalized at time of acute COVID-like symptoms^b^				**0.8**				**0.89**
ICU stay	16 (1.6)	26 (1.2)	0 (0.0)		1 (0.4)	1 (0.2)	0 (0.0)	
Hospitalized (non-ICU)	34 (3.3)	66 (3.1)	2 (5.1)		3 (1.2)	5 (0.8)	0 (0.0)	
Not hospitalized	967 (95.1)	2006 (95.6)	37 (94.9)		243 (98.4)	634 (99.1)	21 (100.0)	

^a^SARS-CoV-2 vaccination status indicates participants with at least one dose prior to the index COVID-19 test; vaccination initiation information was obtained from linked electronic health record data and patient survey responses

^b^Hospitalized for COVID-like symptoms questions not asked on baseline; these questions are asked on each quarterly Follow-up Survey beginning 4-14-2021; the section shown here includes combined data from the 3 mo f/u surveys

Pre-existing conditions question only asked on 3-Month Follow-up Survey beginning 4-14-2021 and have missing data for COVID + (*n* = 51) and COVID − (*n* = 9)

Yellow-labelled cells indicate some cells in the corresponding variable have expected counts < 5 under null hypothesis; all *p*-values are calculated using chi-square test. Chi-square tests were conducted separately within each COVID groups

### Adjusted Marginal Effects of Race and Ethnicity and Gender from GLM

**Fig. 2:**
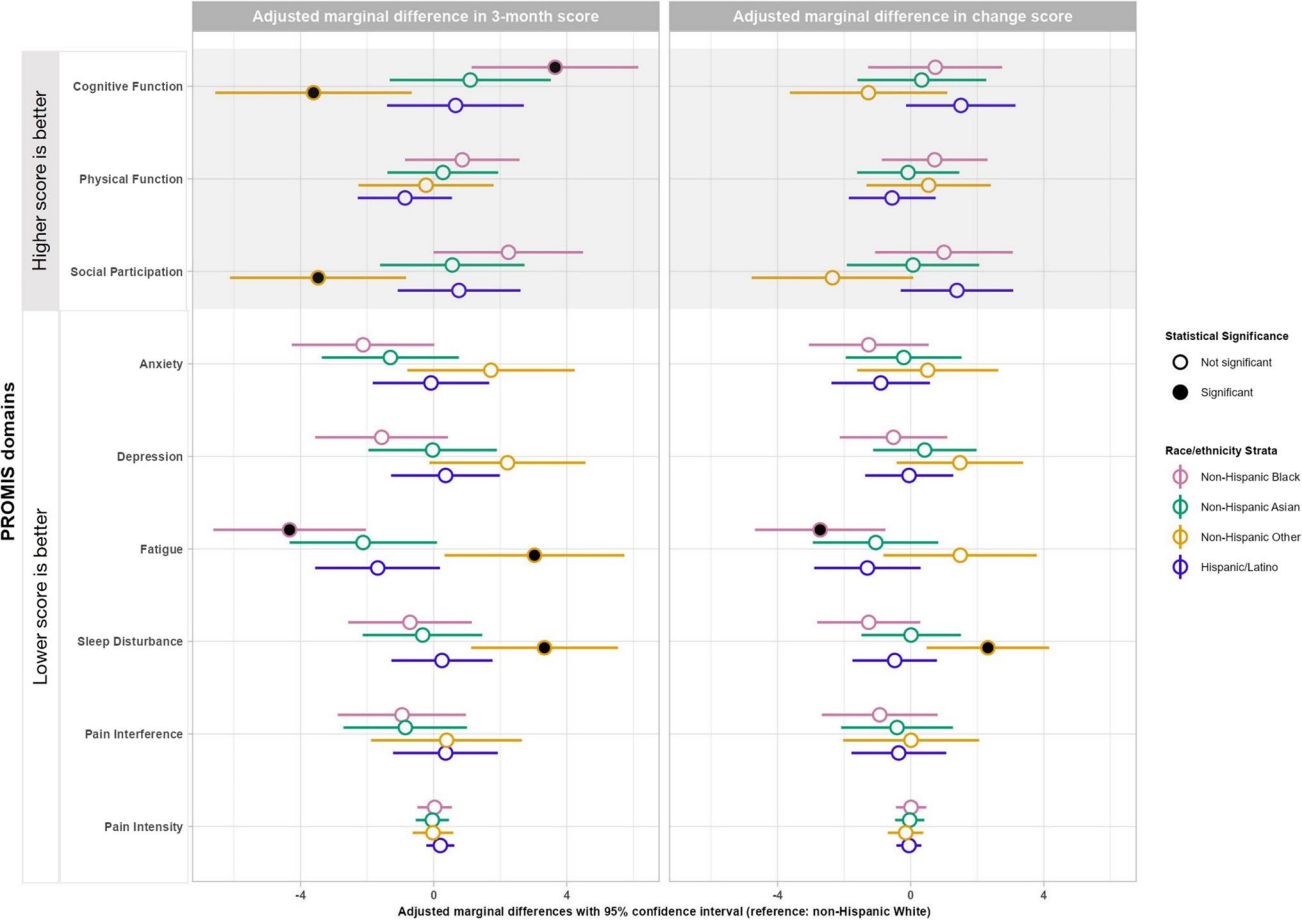
Forest plots of adjusted marginal differences in PROMIS domain *T*-scores and pain intensity scores between the minority racial–ethnic groups and non-Hispanic White in the COVID-positive group

We focused on reporting the adjusted marginal effects (in “estimate [95% confidence interval]” format) of exposure variable groups in the COVID + cohort. Compared with White participants at 3 months, Black participants reported statistically and clinically better cognitive function (3.6 [1.1, 6.2]) and fatigue (− 4.3 [− 6.6, − 2.0]); while Other race group participants reported statistically and clinically worse cognitive function (− 3.6 [− 6.6, − 0.7]), social participation (− 3.5 [− 6.1, − 0.8]), fatigue (3.0 [0.3, 5.7]), and sleep disturbance (3.3 [1.1, 5.5]). In comparing baseline to 3-month outcomes adjusted for baseline PROMIS scores, compared with White participants, Black participants reported more improvement in fatigue (− 2.7 [− 4.7, − 0.8]); Other race group participants reported less improvement in sleep disturbance (2.3 [0.5, 4.2]). There were no other statistically and clinically significant differences across the groups (Table 3, Fig. 2). For COVID- participants, we observed fewer differences. At 3 months, anxiety and sleep disturbance were better for non-Hispanic Black participants, while no differences were found for non-Hispanic Other participants. From baseline to 3 months, cognitive function, anxiety, and sleep disturbance improved more for non-Hispanic Black and physical function improved more for non-Hispanic Asian (Appendix 2).

**Table 3:** Adjusted marginal differences in 3-month PROMIS domain scores and in change scores from baseline to 3-month between the racial-ethnic minoritized groups and non-Hispanic White among COVID + participants

		Adjusted marginal difference (95% CI) Reference = non-Hispanic White			
		Non-Hispanic Black	Non-Hispanic Asian	Hispanic/Latino	Non-Hispanic Other
Adjusted 3-month PROMIS scores					
Higher better	Cognitive function	3.6 (1.1, 6.2)^C^	1.1 (− 1.3, 3.5)	0.7 (− 1.4, 2.7)	− 3.6 (− 6.6, − 0.7)^C^
	Physical function	0.9 (− 0.9, 2.6)	0.3 (− 1.4, 1.9)	− 0.9 (− 2.3, 0.5)	− 0.2 (− 2.3, 1.8)
	Social participation	2.2 (− 0.003, 4.5)^C^	0.6 (− 1.6, 2.7)	0.8 (− 1.1, 2.6)	− 3.5 (− 6.1, − 0.8)^C^
Lower better	Anxiety	− 2.1 (− 4.3, 0.02)^C^	− 1.3 (− 3.4, 0.8)	− 0.1 (− 1.8, 1.7)	1.7 (− 0.8, 4.2)
	Depression	− 1.6 (− 3.6, 0.4)	− 0.0 (− 2.0, 1.9)	0.4 (− 1.3, 2.0)	2.2 (− 0.1, 4.6)^C^
	Fatigue	− 4.3 (− 6.6, − 2.0)^C^	− 2.1 (− 4.3, 0.1)^C^	− 1.7 (− 3.6, 0.2)	3.0 (0.3, 5.7)^C^
	Sleep disturbance	− 0.7 (− 2.6, 1.1)	− 0.3 (− 2.1, 1.5)	0.2 (− 1.3, 1.8)	3.3 (1.1, 5.5)^C^
	Pain interference	− 1.0 (− 2.9, 1.0)	− 0.9 (− 2.7, 1.0)	0.4 (− 1.2, 1.9)	0.4 (− 1.9, 2.6)
	Pain intensity	0.03 (− 0.5, 0.5)	− 0.04 (− 0.5, 0.5)	0.20 (− 0.2, 0.6)	− 0.02 (− 0.6, 0.6)
Change in PROMIS scores additionally adjusted for baseline scores					
Higher better	Cognitive function	0.7 (− 1.3, 2.7)	0.3 (− 1.6, 2.3)	1.5 (− 0.1, 3.1)	− 1.3 (− 3.6, 1.1)
	Physical function	0.7 (− 0.9, 2.3)	− 0.1 (− 1.6, 1.5)	− 0.6 (− 1.9, 0.7)	0.5 (− 1.3, 2.4)
	Social participation	1.0 (− 1.1, 3.1)	0.1 (− 1.9, 2.1)	1.4 (− 0.3, 3.1)	− 2.4 (− 4.8, 0.1)^C^
Lower better	Anxiety	− 1.3 (− 3.1, 0.5)	− 0.2 (− 1.9, 1.5)	− 0.9 (− 2.4, 0.6)	0.5 (− 1.6, 2.6)
	Depression	− 0.5 (− 2.1, 1.1)	0.4 (− 1.1, 2.0)	− 0.0 (− 1.4, 1.3)	1.5 (− 0.4, 3.4)
	Fatigue	− 2.7 (− 4.7, − 0.8)^C^	− 1.1 (− 2.9, 0.8)	− 1.3 (− 2.9, 0.3)	1.5 (− 0.8, 3.8)
	Sleep disturbance	− 1.3 (− 2.8, 0.3)	0.0 (− 1.5, 1.5)	− 0.5 (− 1.7, 0.8)	2.3 (0.5, 4.2)^C^
	Pain interference	− 0.9 (− 2.7, 0.8)	− 0.4 (− 2.1, 1.3)	− 0.4 (− 1.8, 1.1)	0.01 (− 2.0, 2.1)
	Pain intensity	0.01 (− 0.4, 0.5)	− 0.03 (− 0.5, 0.4)	− 0.05 (− 0.4, 0.3)	− 0.2 (− 0.7, 0.4)

(a) The adjusted marginal differences of racial-ethnic minoritized groups compared with the non-Hispanic White participant group in COVID + participants are calculated based on the adjusted estimates from the generalized linear models with adjustment for age, race/ethnicity, gender, education, marital status, health insurance status, family income, employment status, location of baseline testing, tobacco use, pre-existing health conditions, hospitalization, COVID vaccination status in addition to index COVID-19 test result and its interaction with race/ethnicity group variables

(b) Clinical significance indicated by “C” in the superscript. For scores other than pain intensity, a difference in score of ≥ 2 is considered clinically significant and for pain intensity, a score difference of ≥ 1 is considered clinically significant

**Fig. 3:**
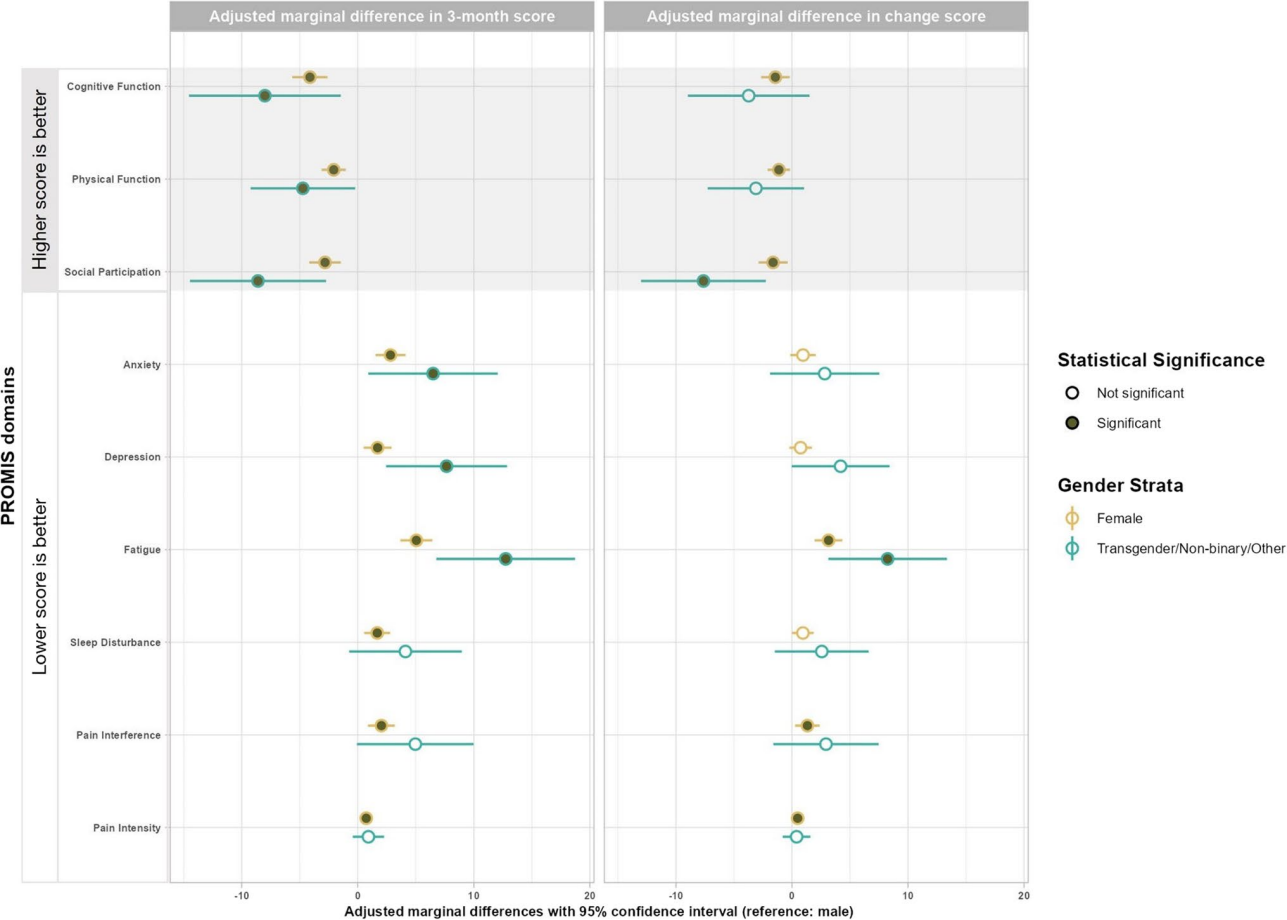
Forest plots of adjusted marginal differences in PROMIS domain *T*-scores and pain intensity scores between the gender groups and male in the COVID-positive group

Among COVID + participants, compared with males, females reported worse cognitive function (− 4.1 [− 5.6, − 2.6]), physical function (− 2.1 [− 3.1, − 1.0]), social participation (− 2.8 [− 4.2, − 1.5]), anxiety (2.8 [1.5, 4.1]), fatigue (5.1 [3.7, 6.4]), and pain interference (2.0 [0.9, 3.2]). Transgender/non-binary/other gender participants had worse scores for cognitive function (− 8.0 [− 14.5, − 1.5]), physical function (− 4.7 [− 9.2, − 0.2]), social participation (− 8.6 [− 14.5, − 2.7]), anxiety (6.5 [0.9, 12.1]), depression (7.7 [2.4, 12.9]), and fatigue (12.8 [6.8, 18.7]). In addition, females reported less improvement for fatigue (3.1 [2.0, 4.3]), and the transgender/non-binary/other group reported less improvement for social participation (− 7.6 [− 13.0, − 2.3]) and fatigue (8.2 [3.1, 13.4]) over the 3 months. There were no other statistically and clinically significant differences across the groups (Table 4, Fig. 3). For COVID − participants, compared with males, female participants reported more sleep disturbance at 3 months, while the transgender/non-binary group reported worse across all domains. When examining changes over the 3 months, physical function and social participation were less improved for females, while physical function, anxiety, sleep disturbance, and pain interreference were less improved for transgender/non-binary participants (Appendix 3).

**Table 4:** Adjusted marginal differences in 3-month PROMIS domain scores and in change scores from baseline to 3-month between gender groups among COVID + participants

		Adjusted marginal difference (95% CI) Reference = male	
		Female	Transgender/non-binary/Other
3-month PROMIS scores			
Higher better	Cognitive function	− 4.1 (− 5.6, − 2.6)^C^	− 8.0 (− 14.5, − 1.5)^C^
	Physical function	− 2.1 (− 3.1, − 1.0)^C^	− 4.7 (− 9.2, − 0.2)^C^
	Social participation	− 2.8 (− 4.2, − 1.5)^C^	− 8.6 (− 14.5, − 2.7)^C^
Lower better	Anxiety	2.8 (1.5, 4.1)^C^	6.5 (0.9, 12.1)^C^
	Depression	1.7 (0.5, 2.9)	7.6 (2.4, 12.9)^C^
	Fatigue	5.1 (3.7, 6.4)^C^	12.7 (6.8, 18.7)^C^
	Sleep disturbance	1.7 (0.5, 2.8)	4.1 (− 0.7, 9.0)^C^
	Pain interference	2.0 (0.9, 3.2)^C^	5.0 (− 0.1, 10.0)^C^
	Pain intensity	0.7 (0.4, 1.0)	0.9 (− 0.4, 2.3)
Change in PROMIS scores additionally adjusted for baseline scores			
Higher better	Cognitive function	− 1.4 (− 2.7, − 0.2)	− 3.7 (− 9.0, 1.5)^C^
	Physical function	− 1.1 (− 2.1, − 0.2)	− 3.1 (− 7.3, 1.0)^C^
	Social participation	− 1.6 (− 2.9, − 0.4)	− 7.6 (− 13.0, − 2.3)^C^
Lower better	Anxiety	0.9 (− 0.2, 2.0)	2.8 (− 1.9, 7.5)^C^
	Depression	0.7 (− 0.2, 1.7)	4.2 (− 0.02, 8.4)^C^
	Fatigue	3.1 (2.0, 4.3)^C^	8.2 (3.1, 13.4)^C^
	Sleep disturbance	0.9 (− 0.004, 1.9)	2.6 (− 1.5, 6.6)^C^
	Pain interference	1.3 (0.3, 2.4)	2.9 (− 1.6, 7.5)^C^
	Pain intensity	0.5 (0.2, 0.8)	0.4 (− 0.8, 1.6)

(a) The adjusted marginal differences of gender groups comparing with the non-Hispanic White participant group in COVID + participants are calculated based on the adjusted estimates from the generalized linear models with adjustment for age, race/ethnicity, gender, education, marital status, health insurance status, family income, employment status, location of baseline testing, tobacco use, pre-existing health conditions, hospitalization, COVID vaccination status in addition to index COVID-19 test result and its interaction with gender group variables

(b) Clinical significance indicated by “C” in the superscript. For scores other than pain intensity, a difference in score of ≥ 2 is considered clinically significant and for pain intensity, a score difference of ≥ 1 is considered clinically significant

## Discussion

The COVID-19 pandemic exacerbated existing racial, ethnic, and gender disparities in the US, as highlighted by differences in risk of initial SARS-CoV-2 infection, risk of morbidity and mortality from COVID-19, and risk of longer term symptoms. Wisk et al. found improvements in reports of well-being relative to social participation among participants who were COVID + compared to those who were COVID − ; setting the stage for this authorship team to further explore whether improvements by race, ethnicity, and gender existed across other domains of well-being.

In our earlier research, we found statistically and clinically significant improvements in well-being for participants in the COVID + group vs. the COVID − group for social participation, but not for changes in other well-being domains [7]. It is worth noting that these results were measured in models accounting for multiple patient characteristics such as income, education, pre-existing health conditions, and severity of illness, suggesting that biological and societal factors beyond these observable factors may have a role in the differences we observed. Although this study did not control for all social determinants of health (SDOH) and related variables, it extends the work with a larger sample focused more closely on differences in PROMIS-related outcomes by race, ethnicity, and gender.

Our findings add to prior research that found that racial and ethnic minoritized populations were at higher risk of infection [1–3] and higher risk of severe infection by adding measures of overall health and well-being [1, 27]. Correlations found in this secondary analysis highlight several inequities separate from those previously described [2–5]. The PROMIS-29 tool, a validated holistic measure of health and well-being, at time of acute illness and at 3 months later allowed us to explore potential differences across racial, ethnic, and gender populations in lived experiences with COVID-19. Use of PROMIS-29 to assess whether race, ethnicity, and/or gender differences in self-reports of well-being and quality of life existed at 3 months after having symptoms of an acute COVID-19 illness and recovery over a 3-month period (from baseline to 3 months) informed how participants may have perceived their experience with clinical and social factors that may have impacted their recovery from COVID-19. Differences by race and ethnicity in the self-evaluation of well-being utilizing PROMIS-29 scores at 3 months and recovery over a 3-month period after the acute illness within cohorts that tested positive for COVID-19 were identified and have potential to translate to clinical differences in the population.

Improvements across racial and ethnic groups with well-being relative to physical function, social participation, fatigue, and pain interference within the COVID + group complement the findings of racial and ethnic differences in disease severity and hospitalization rates. Price-Haywood and colleagues (2020) assessed clinical outcomes from a large cohort of COVID + patients in Louisiana and found that Black patients who had a higher prevalence of hospitalizations also had a higher prevalence of chronic diseases (e.g., obesity) than White patients, which were likely due to underlying chronic conditions [28]. Similarly, Black participants in the INSPIRE study had a higher rate of pre-existing conditions, including asthma and obesity, compared to White participants. Price-Haywood and colleagues (2020) described the multifactorial reasons for racial differences, citing SDOH factors like job type, lower education, and lower income levels [28]. Acute COVID-19 disproportionately impacted race/ethnic minoritized groups; yet, these groups do not appear to be disproportionately impacted as it relates to PROMIS-29 measures among participants in our study. It is likely that race and ethnic minoritized populations have a baseline purview of their personal health and well-being is inextricably linked to a normalcy of coping with pre-existing health conditions and an array of SDOH that include oppression, injustice, and often times, poverty [29]. When comparing Black and White adult Americans on the effects of self-rated health, disjointed links between health and happens were hypothesized as being due to different racial, ethnic, and cultural perceptions of physical health in addition to salience of physical health as an aspect of perceived happiness. The lived experience of race and ethnic minoritized groups may require an adaptative response to centuries of health-related adversities resulting from the intersection of SDOH and may influence self-perceptions of personal health and well-being when compared to their White counterparts. These fundamental differences in may contribute to what seems like a contradiction between racial and ethnic groups relative to their self-rated health using PROMIS. While our previous work examining our entire cohort found improvements only in social participation, we found that stratification by race and ethnicity revealed statistically significant improvements in cognitive function and fatigue among select participant groups, suggesting the need for analyses specific to race and ethnicity when assessing well-being among individuals with a previous or current COVID + status [7].

The PROMIS-29 has been used to measure the impacts of health conditions on physical and mental health function according to gender, such as differences by gender in physical function and experiences with pain [30, 31]. Numerous other studies have identified gender-based differences in PROMIS-29 well-being measures following acute illness and injury, suggesting female gender has been consistently associated reports of larger decrements than men but may return to similar functionality over time [6, 32]. While SDOH may be driving the gender differences, using gender to help risk stratify is pragmatic and potentially needed.

As with race and ethnicity, there is a paucity of literature evaluating long-term COVID-19 post-acute symptoms by gender, and overall health and well-being during the post-acute period have received even less attention. Prior to the COVID-19 pandemic, health disparities between males, females, and non-binary/trans/other groups were established in the literature. In a study investigating SDOH in women and men, findings indicated that lower health among females compared to males were associated with education, household economic status, employment, and marital status [33]. Similarly, responses from a 2016 community-driven survey some gay, queer, and nonbinary individuals have reported significantly worse self-reported health status compared to binary trans individuals [34]. Worse self-reports were correlated to SDOH that include a lower education level, more economic stress, and/or belonging to an ethnic, religious, sexual, or ability minoritized group. Together, an alignment with worse health among gender minoritized populations appear to be linked to similar SDOH. The COVID-19 pandemic seems to have exacerbated those existing health disparities. Jacobs and colleagues (2020) assessed gender differences among 183 participants who reported COVID-19-related symptoms, revealing that women with more severe disease had higher odds of disease persistence at 35 days and negative impact on their quality-of-life estimates related to daily function [35]. Jevotovsky and colleagues (2021) did not identify significant differences by gender with PROMIS scores in their retrospective review of how age and gender were related to PROMIS-29 scores in patients. Jacobs and colleagues (2020) cautions that the potential for gender-based differences impacting quality of life, health, physical, and mental function cannot be ruled out and warrants further evaluation after an acute illness [35]. Comparatively, in our INSPIRE cohort, gender contributed to significant differences between baseline and 3 months when comparing subgroups of females and transgender to the male populations. Specifically, females scored worse at 3 months in cognitive function, physical function, social participation, anxiety, fatigue, and pain interference when compared to males; and when exploring changes from baseline to 3 months, females had less improvement in fatigue than males. Our findings are consistent with a similar study where females were statistically significantly more likely to experience fatigue than males when sex differences in reported symptoms up to 5 months following an acute COVID-19 illness were explored [36]. Overall, findings of that study showed that females were more symptomatic than males not only in the acute phase, but also during the follow-up period, which may warrant future exploration. These research findings add to the sparse literature describing gender differences after an acute illness with COVID-19.

Gender differences between symptoms reported between male and female participants could stem from the disproportionate burdens placed upon females at the societal level amidst the COVID-19 pandemic. Gaps in unemployment and labor force participation increased between men and women during the COVID-19 pandemic, and were particularly noticeable during the summer months in 2020 [37]. Female healthcare workers experienced a higher proportion of anxiety than their male counterparts [38]. Furthermore, as females are typically more often the primary caregiver in the household, even females who work outside of the home experienced an increase in time spent performing household tasks and caring for children and parents when compared to their male counterparts and colleagues [39]. The increase in household related responsibilities was exacerbated by societal changes in response to the pandemic that included new work-from-home requirements while child care facilities were closed and school-aged children were expected to engage in distance learning [40]. For the transgender community, the COVID-19 pandemic disrupted access to gender-affirming care, decreased access to healthcare, and exacerbated existing economic disparities [41, 42]. These significant changes in societal expectations and changes in gender-based expectations may help explain differences in health and well-being by gender reported by our participants. Our findings are consistent with mechanisms of both biological sex and gender identity having potential to be significant predictors of persistent symptoms after an acute COVID-19 illness, including both physical and psychosocial symptoms. Future studies should explore how these factors, both at the individual and intersectional levels, may be driving the gender differences found in this secondary analysis.

While there were only 27 participants in the COVID + and 15 in the COVID − group of transgender/non-binary/other participants, the research team deemed inclusion of this group of participants in the analysis as necessary and worthwhile to report given the limited amount of long COVID-19-related research available for this understudied population. This subgroup of COVID + participants had worse scores for cognitive function, physical function, social participation, anxiety, depression, and fatigue than males at 3 months. When assessing changes over 3 months from baseline to 3 months, this subgroup experienced less improvement for social participation and fatigue. Significant differences in psychosocial challenges between LGBTQ + community members, specifically transgender/non-binary individuals in the INSPIRE study, resonate with social and health factors that hinder well-being, including prolonged social isolation, social stigma, and the stresses of being a member of a gender minoritized group [43, 44]. Ghabrial and colleagues (2023) explored the effect of changes in access to peer gatherings for trans and non-binary people on anxiety and depression during the COVID-19 pandemic and found that of 780 participants, most reported a negative impact on access to peer gatherings during COVID-19 along with an increase in depression and anxiety symptoms from pre-pandemic to follow-up [45]. Findings of COVID + transgender and non-binary participants in the INSPIRE study support research findings that shed light on the psychosocial impact of COVID-19 on the well-being of this population, and illustrate the need to address barriers to health equity for LGBTQ + community members.

Overall, our analysis revealed some differences by race/ethnicity and gender in psychosocial and physical symptoms both at 3 months and the period between baseline and 3 months. The persistence of COVID-19 symptoms after acute illness seen in females and transgender/non-binary, and other gender identifying groups suggests a possible inequity in mental health for these groups [45]. Our study findings highlight a potential need for focus and resource allocation to mental health and well-being management for females and transgender/non-binary, and other gender identifying groups after experiencing an acute COVID-19 illness.

## Limitations

In addition to several study strengths, [7] the study also has several limitations. First, we sought to recruit and engage a diverse population across the US with requirements of a verifiable COVID-19 test, an existing electronic health record (EHR), and access to a working internet-enabled device to complete study components (i.e., online surveys). These requirements may have biased the sample to exclude populations who lacked internet and EHRs, as well as those who were unable or unwilling to participate. The potential for excluding community members with a low socioeconomic status (SES) might have a more extensive impact on recruitment of race/ethnic and gender minoritized participants that might be at low SES populations if the marginal benefit of compensation was higher among people with lower income. Furthermore, unstable income or loss of employment, or other changes in financial status over the 3-month period could have contributed to failure to complete the 3-month survey for some INSPIRE participants.

Second, our findings could be subject to non-response bias because a significant proportion (25%) of eligible respondents did not complete a 3-month survey and there were differences in response rates by race/ethnicity. In relation to retention, the study experienced a differential drop out by some race/ethnicity groups, namely non-Hispanic Black participants dropped out quite a bit more than other populations. This might have based that group, leading to a lack of findings that might have been there if we had better equity in retention across enrolled participants. Third, differences in symptoms during an acute COVID-19 illness (i.e., varying symptoms with varying severity) makes it challenging to assess whether COVID − participants would be expected to experience more or less severe symptoms over time. In addition, comparison of COVID + participants to the COVID − group could underestimate changes in well-being versus if we compared COVID + participants to the general population who did not experience illness. Fourth, COVID-19 tests may yield false-negative or false-positive results; therefore, we cannot exclude the possibility that participants may have been misclassified to a COVID status based on their test result. This misclassification could bias the understanding gleaned from changes in well-being observed between groups. However, the tests used with enrolled participants were fairly robust and well verified, as test results were a required inclusion criteria. Fifth, a 3-month follow-up post-baseline report of COVID-like symptoms is only one short-term observation of changes in well-being; however, this analytic approach reduces the likelihood of biases that may result from the absence of standardization. Sixth, several notable characteristic differences are present between the groups (Tables 1 and 2). However, pairwise testing was not performed between variables, and comparisons were not made to the reference group as these variables were not an outcome of our study. These differences raise additional questions though that may serve to direct future study. Seventh, the findings of better outcomes for Hispanic and Latino patients in combination of worse outcomes for women raises the question of possible interaction between race, ethnicity, and gender. While all analyses were adjusted for race, ethnicity, and gender, future studies could benefit from focusing on the intersectionality between race, ethnicity, and gender.

## Conclusion

We found that, across a national, multi-site prospective registry, Black participants reported better outcomes at 3 months after a COVID + illness relative to White participants in cognitive function, social participation, anxiety, and fatigue; with no significant differences noted in other domains (physical function, depression, sleep disturbance, pain interference, or pain intensity). Reports of well-being were worse for females and transgender/non-binary/other gender participants at 3 months when compared to male participants. Given race/ethnicity, and gender-based differences, future race, ethnic, and gender-based studies are warranted to provide clarity and insight as to why these differences exist.

## Supplementary Information

Below is the link to the electronic supplementary material.

Supplementary file1 (DOCX 24 KB)

Supplementary file2 (DOCX 24 KB)

Supplementary file3 (DOCX 27 KB)

Supplementary file4 (DOCX 38 KB)

Supplementary file5 (DOCX 38.3 KB)

## Data Availability

The datasets presented in this article are not readily available because the data are not approved for outside use. Requests to access the datasets should be directed to Mandy Hill at mandy.j.hill@uth.tmc.edu.
