# Supplementary material for: Race, Ethnicity, and Gender Differences in Patient Reported Well-Being and Cognitive Functioning Within 3 Months of Symptomatic Illness During COVID-19 Pandemic
Source: J Racial Ethn Health Disparities. 2024 Aug 22;12(5):3192–209. doi: 10.1007/s40615-024-02124-8 (PMC11891493; doi:10.1007/s40615-024-02124-8)
Supplement: Supplementary file 2 — Supplementary file2 (DOCX 24 KB) [file 40615_2024_2124_MOESM2_ESM.docx]

**Appendix 1b.** Adjusted Marginal Difference in Change in Score Estimation from the GLM

Example of calculating the marginal gender effects on change in each PROMIS outcomes from baseline to 3-month:

Using GLM, the regression on change scores from baseline to 3 months of each PROMIS domain or pain intesity is calculated as follows:

$${(change)}_{i}=\beta_{0}+{female}_{i}*\beta_{1}+{other}_{i}*\beta_{2}+ {covid}_{i}*\beta_{3}+ {(female}_{i}*{covid}_{i}){*\beta}_{4}+ {(other}_{i}*{covid}_{i}){*\beta}_{5} + {BSScore}_{i}{*\beta}_{6}+ {\boldsymbol{X}_{\boldsymbol{i}}}^{\boldsymbol{'}}*\boldsymbol{B}$$

Where $\boldsymbol{BSScore}_{\boldsymbol{i}}$ is the corresponding baseline PROMIS score, $\boldsymbol{X}_{\boldsymbol{i}}$ denotes a vector of observed values of independent variables for patient $i$, $\boldsymbol{B}$ denotes these covariates’ coefficients for outcome (e.g. physical function) and,

$${covid}_{i}=\left\{ \begin{aligned} 1, &if COVID+ \\ 0, &else \end{aligned} \right.$$

$${female}_{i}=\left\{ \begin{aligned} 1, &if gender is female \\ 0, &else \end{aligned} \right.$$

$${other}_{i}=\left\{ \begin{aligned} 1, &if gender is transgender/non-binary other \\ 0, &else \end{aligned} \right.$$

The estimated coefficients (hated-betas) from the GLM are used to calculate the adjusted marginal difference in change-score. For example, the adjusted marginal difference in change-score (from baseline to 3-months follow-up) for physical function of female covid-positive participants in comparison to the change-score for physical function of male covid-positive participants is calculated as below:

$${{change}_{physical\_function}\left( female vs. male \right)|}_{COVID+}=\hat{\beta}_{1}+ \hat{\beta}_{4}$$
