# Supplementary material for: Race, Ethnicity, and Gender Differences in Patient Reported Well-Being and Cognitive Functioning Within 3 Months of Symptomatic Illness During COVID-19 Pandemic
Source: J Racial Ethn Health Disparities. 2024 Aug 22;12(5):3192–209. doi: 10.1007/s40615-024-02124-8 (PMC11891493; doi:10.1007/s40615-024-02124-8)
Supplement: Supplementary file 3 — Supplementary file3 (DOCX 27 KB) [file 40615_2024_2124_MOESM3_ESM.docx]

**Appendix 2.** Adjusted marginal differences in 3-month PROMIS domain scores and differences in change in scores from baseline to 3-month in race/ethnicity groups among COVID- participants

|  |  | **Adjusted Marginal Difference (95% CI)**  **Reference = Non-Hispanic White** | | | |
| --- | --- | --- | --- | --- | --- |
|  |  | Non-Hispanic Black | Non-Hispanic Asian | Hispanic/Latino | Non-Hispanic Other |
| Adjusted 3-Month PROMIS Scores | | | | | |
| Higher Better | Cognitive Function | 5.13 (1.51, 8.75)^C^ | 0.39 (-2.84, 3.63) | 0.76 (-2.33, 3.85) | -1.65 (-7.53, 4.23) |
|  | Physical Function | 2.33 (-0.16, 4.83)^C^ | 3.37 (1.14, 5.59)^C^ | -0.55 (-2.68, 1.57) | 0.73 (-3.32, 4.77) |
|  | Social Participation | 2.92 (-0.33, 6.17)^C^ | 0.26 (-2.64, 3.16) | -0.87 (-3.65, 1.90) | -3.93 (-9.20, 1.35)^C^ |
| Lower Better | Anxiety | -3.39 (-6.48, -0.31)^C^ | -2.50 (-5.26, 0.25)^C^ | -0.41 (-3.04, 2.22) | 3.36 (-1.66, 8.37)^C^ |
|  | Depression | -1.90 (-4.78, 0.98) | -0.93 (-3.51, 1.64) | -1.74 (-4.20, 0.72) | 2.49 (-2.20, 7.17)^C^ |
|  | Fatigue | -4.70 (-8.01, -1.39)^C^ | -2.87 (-5.82, 0.09)^C^ | -2.39 (-5.22, 0.44)^C^ | 3.71 (-1.67, 9.09)^C^ |
|  | Sleep Disturbance | -3.31 (-6.00, -0.63)^C^ | 0.25 (-2.14, 2.65) | -1.08 (-3.37, 1.20) | -0.10 (-4.46, 4.26) |
|  | Pain Interference | -0.55 (-3.33, 2.23) | -1.91 (-4.40, 0.57) | -0.30 (-2.67, 2.07) | 2.40 (-2.12, 6.91)^C^ |
|  | Pain Intensity | 0.56 (-0.18, 1.31) | -0.50 (-1.17, 0.17) | 0.01 (-0.63, 0.65) | -0.19 (-1.40, 1.03) |
| Change in PROMIS scores additionally adjusted for baseline scores | | | | | |
| Higher Better | Cognitive Function | 4.16 (1.27, 7.05)^C^ | 0.77 (-1.81, 3.36) | -0.43 (-2.90, 2.04) | 0.10 (-4.60, 4.80) |
|  | Physical Function | 2.15 (-0.14, 4.45)^C^ | 2.69 (0.63, 4.74)^C^ | -0.28 (-2.24, 1.68) | 0.51 (-3.22, 4.24) |
|  | Social Participation | 2.97 (-0.00, 5.95)^C^ | 0.27 (-2.39, 2.93) | -0.38 (-2.92, 2.16) | -4.36 (-9.19, 0.47)^C^ |
| Lower Better | Anxiety | -2.66 (-5.26, -0.06)^C^ | -2.05 (-4.37, 0.27)^C^ | 0.16 (-2.06, 2.38) | 2.04 (-2.19, 6.26)^C^ |
|  | Depression | -2.03 (-4.36, 0.30)^C^ | -0.41 (-2.49, 1.67) | 0.13 (-1.87, 2.12) | 1.54 (-2.24, 5.33) |
|  | Fatigue | -4.44 (-7.27, -1.62)^C^ | -2.27 (-4.79, 0.25)^C^ | -1.48 (-3.89, 0.93) | 5.09 (0.50, 9.68)^C^ |
|  | Sleep Disturbance | -2.71 (-4.95, -0.47)^C^ | 0.41 (-1.59, 2.41) | -0.86 (-2.77, 1.05) | 0.39 (-3.25, 4.02) |
|  | Pain Interference | -0.43 (-2.94, 2.08) | -1.08 (-3.32, 1.16) | -0.61 (-2.75, 1.53) | 2.97 (-1.11, 7.05)^C^ |
|  | Pain Intensity | 0.32 (-0.34, 0.98) | -0.40 (-0.99, 0.20) | -0.25 (-0.81, 0.32) | -0.03 (-1.10, 1.04) |

Note: (a) The adjusted marginal differences of racial-ethnic minoritized groups compared with the non-Hispanic White participant group in COVID- participants are calculated based on the adjusted estimates from the generalized linear models with adjustment for age, race/ethnicity, gender, education, marital status, health insurance status, family income, employment status, location of baseline testing, tobacco use, pre-existing health conditions, hospitalization, COVID vaccination status in addition to index COVID-19 test result and its interaction with race/ethnicity group variables.

(b)Clinical significance indicated by ‘C’ in the superscript. For scores other than pain intensity, a difference in score of ≥2 is considered clinically significant and for pain intensity, a score difference of ≥ 1 is considered clinically significant.
