# Supplementary material for: Race, Ethnicity, and Gender Differences in Patient Reported Well-Being and Cognitive Functioning Within 3 Months of Symptomatic Illness During COVID-19 Pandemic
Source: J Racial Ethn Health Disparities. 2024 Aug 22;12(5):3192–209. doi: 10.1007/s40615-024-02124-8 (PMC11891493; doi:10.1007/s40615-024-02124-8)
Supplement: Supplementary file 4 — Supplementary file4 (DOCX 38 KB) [file 40615_2024_2124_MOESM4_ESM.docx]

**Appendix 3**. Adjusted marginal differences in 3-month PROMIS domain scores and differences in change in scores from baseline to 3-month in gender groups among COVID- participants

|  |  | **Adjusted Marginal Difference (95% CI)**  **Reference = Male** | |
| --- | --- | --- | --- |
|  |  | Female | Transgender/Non-binary/Other |
| 3-Month PROMIS Scores | | | |
| Higher Better | Cognitive Function | -3.77 (-6.26, -1.29)^C^ | -8.13 (-14.24, -2.02)^C^ |
|  | Physical Function | -2.92 (-4.63, -1.20)^C^ | -7.66 (-11.87, -3.44)^C^ |
|  | Social Participation | -4.52 (-6.75, -2.29)^C^ | -9.26 (-14.74, -3.79)^C^ |
| Lower Better | Anxiety | 2.43 (0.31, 4.55)^C^ | 8.24 (3.04, 13.45)^C^ |
|  | Depression | 1.57 (-0.41, 3.54) | 6.68 (1.81, 11.55)^C^ |
|  | Fatigue | 4.55 (2.28, 6.82)^C^ | 11.12 (5.53, 16.71)^C^ |
|  | Sleep Disturbance | 2.17 (0.32, 4.01)^C^ | 5.94 (1.41, 10.48)^C^ |
|  | Pain Interference | 2.32 (0.42, 4.23)^C^ | 6.65 (1.96, 11.34)^C^ |
|  | Pain Intensity | 0.71 (0.20, 1.22) | 1.29 (0.03, 2.55)^C^ |
| Change in PROMIS scores additionally adjusted for baseline scores | | | |
| Higher Better | Cognitive Function | -1.16 (-3.16, 0.84) | -2.14 (-7.05, 2.77)^C^ |
|  | Physical Function | -2.31 (-3.90, -0.73)^C^ | -5.54 (-9.43, -1.65)^C^ |
|  | Social Participation | -3.30 (-5.35, -1.26)^C^ | -5.33 (-10.38, -0.29)^C^ |
| Lower Better | Anxiety | -0.06 (-1.86, 1.74) | 4.56 (0.16, 8.96)^C^ |
|  | Depression | -0.29 (-1.89, 1.31) | 1.66 (-2.29, 5.61) |
|  | Fatigue | 2.15 (0.20, 4.10)^C^ | 5.33 (0.53, 10.12)^C^ |
|  | Sleep Disturbance | 1.96 (0.42, 3.50) | 3.99 (0.20, 7.77)^C^ |
|  | Pain Interference | 1.52 (-0.21, 3.24) | 5.08 (0.84, 9.32)^C^ |
|  | Pain Intensity | 0.51 (0.05, 0.96) | 1.10 (-0.02, 2.21)^C^ |

Note: (a) The adjusted marginal differences of gender groups compared with the non-Hispanic White participant group in COVID- participants are calculated based on the adjusted estimates from the generalized linear models with adjustment for age, race/ethnicity, gender, education, marital status, health insurance status, family income, employment status, location of baseline testing, tobacco use, pre-existing health conditions, hospitalization, COVID vaccination status in addition to index COVID-19 test result and its interaction with gender group variables.

(b)Clinical significance indicated by ‘C’ in the superscript. For scores other than pain intensity, a difference in score of ≥2 is considered clinically significant and for pain intensity, a score difference of ≥ 1 is considered clinically significant.
