# Supplementary material for: Race, Ethnicity, and Gender Differences in Patient Reported Well-Being and Cognitive Functioning Within 3 Months of Symptomatic Illness During COVID-19 Pandemic
Source: J Racial Ethn Health Disparities. 2024 Aug 22;12(5):3192–209. doi: 10.1007/s40615-024-02124-8 (PMC11891493; doi:10.1007/s40615-024-02124-8)
Supplement: Supplementary file 5 — Supplementary file5 (DOCX 38.3 KB) [file 40615_2024_2124_MOESM5_ESM.docx]

**Appendix 4.** Model fit statistics (R^2^ measures) for the adjusted models

| **Model Details** | **Outcome** | **Model R^2^** |
| --- | --- | --- |
| Outcomes analyzed for race-ethnicity at 3 months | Cognitive Function | 0.12 |
|  | Physical Function | 0.25 |
|  | Social Participation | 0.14 |
|  | Anxiety | 0.14 |
|  | Depression | 0.11 |
|  | Fatigue | 0.17 |
|  | Sleep Disturbance | 0.15 |
|  | Pain Interference | 0.21 |
|  | Pain Intensity | 0.23 |
| Change in outcomes analyzed for Race-ethnicity at 3 months with additional baseline adjustment | Cognitive Function | 0.22 |
|  | Physical Function | 0.48 |
|  | Social Participation | 0.39 |
|  | Anxiety | 0.25 |
|  | Depression | 0.23 |
|  | Fatigue | 0.24 |
|  | Sleep Disturbance | 0.27 |
|  | Pain Interference | 0.39 |
|  | Pain Intensity | 0.33 |
| Outcomes analyzed for gender at 3 months | Cognitive Function | 0.12 |
|  | Physical Function | 0.25 |
|  | Social Participation | 0.14 |
|  | Anxiety | 0.14 |
|  | Depression | 0.11 |
|  | Fatigue | 0.17 |
|  | Sleep Disturbance | 0.15 |
|  | Pain Interference | 0.21 |
|  | Pain Intensity | 0.23 |
| Change in outcomes analyzed for gender at 3 months with additional baseline adjustment | Cognitive Function | 0.22 |
|  | Physical Function | 0.48 |
|  | Social Participation | 0.39 |
|  | Anxiety | 0.25 |
|  | Depression | 0.23 |
|  | Fatigue | 0.24 |
|  | Sleep Disturbance | 0.27 |
|  | Pain Interference | 0.39 |
|  | Pain Intensity | 0.33 |

Note: All models utilized **identity link functions** assuming **Gaussian** family of errors. Each model had adjustment for age, race/ethnicity or gender, education, marital status, health insurance status, family income, employment status, location of baseline testing, tobacco use, pre-existing health conditions, hospitalization, COVID vaccination status in addition to index COVID-19 test result and its interaction with the exposure variables.

**Appendix 5.** Adjusted marginal differences in 3-month PROMIS domain scores and in change scores from baseline to 3-month between the racial-ethnic minoritized groups and Non-Hispanic White among COVID+ participants reported with p-values

|  |  | **Estimates from Adjusted Marginal Difference with P-values** | | | | | | | |
| --- | --- | --- | --- | --- | --- | --- | --- | --- | --- |
|  |  | **Reference = Non-Hispanic White** | | | | | | | |
|  |  | Non-Hispanic Black | | Non-Hispanic Asian | | Hispanic/Latino | | Non-Hispanic Other | |
|  |  | Estimate | P-value | Estimate | P-value | Estimate | P-value | Estimate | P-value |
| Adjusted 3-Month PROMIS Scores | | | | | | | | | |
| Higher Better | Cognitive Function | 3.65 | 0.004 | 1.1 | 0.373 | 0.66 | 0.53 | -3.61 | 0.017 |
|  | Physical Function | 0.86 | 0.328 | 0.28 | 0.743 | -0.86 | 0.23 | -0.23 | 0.827 |
|  | Social Participation | 2.25 | 0.05 | 0.56 | 0.613 | 0.76 | 0.417 | -3.47 | 0.01 |
| Lower Better | Anxiety | -2.12 | 0.052 | -1.3 | 0.216 | -0.08 | 0.931 | 1.72 | 0.178 |
|  | Depression | -1.56 | 0.125 | -0.03 | 0.976 | 0.36 | 0.668 | 2.22 | 0.065 |
|  | Fatigue | -4.33 | <0.001 | -2.12 | 0.061 | -1.68 | 0.079 | 3.03 | 0.028 |
|  | Sleep Disturbance | -0.71 | 0.454 | -0.33 | 0.715 | 0.25 | 0.748 | 3.33 | 0.003 |
|  | Pain Interference | -0.95 | 0.331 | -0.85 | 0.367 | 0.36 | 0.657 | 0.39 | 0.738 |
|  | Pain Intensity | 0.03 | 0.906 | -0.04 | 0.886 | 0.2 | 0.357 | -0.02 | 0.955 |
| Change in PROMIS scores additionally adjusted for baseline scores | | | | | | | | | |
| Higher Better | Cognitive Function | 0.74 | 0.473 | 0.33 | 0.734 | 1.51 | 0.072 | -1.27 | 0.293 |
|  | Physical Function | 0.72 | 0.378 | -0.08 | 0.922 | -0.56 | 0.402 | 0.54 | 0.571 |
|  | Social Participation | 1 | 0.342 | 0.07 | 0.945 | 1.39 | 0.106 | -2.35 | 0.058 |
| Lower Better | Anxiety | -1.26 | 0.17 | -0.21 | 0.816 | -0.9 | 0.232 | 0.51 | 0.639 |
|  | Depression | -0.52 | 0.531 | 0.42 | 0.593 | -0.05 | 0.945 | 1.48 | 0.126 |
|  | Fatigue | -2.72 | 0.006 | -1.05 | 0.274 | -1.3 | 0.112 | 1.49 | 0.206 |
|  | Sleep Disturbance | -1.26 | 0.111 | 0.01 | 0.987 | -0.48 | 0.462 | 2.32 | 0.014 |
|  | Pain Interference | -0.93 | 0.293 | -0.41 | 0.632 | -0.36 | 0.623 | 0.01 | 0.99 |
|  | Pain Intensity | 0.01 | 0.958 | -0.03 | 0.905 | -0.05 | 0.778 | -0.15 | 0.576 |

Note: The adjusted marginal differences of racial-ethnic minoritized groups compared with the non-Hispanic White participant group in COVID+ participants are calculated based on the adjusted estimates from the generalized linear models with adjustment for age, race/ethnicity, gender, education, marital status, health insurance status, family income, employment status, location of baseline testing, tobacco use, pre-existing health conditions, hospitalization, COVID vaccination status in addition to index COVID-19 test result and its interaction with race/ethnicity group variables.

**Appendix 6.** Adjusted marginal differences in 3-month PROMIS domain scores and in change scores from baseline to 3-month between gender groups among COVID+ participants reported with p-values

|  |  | **Estimates from Adjusted Marginal Difference with P-values** | | | |
| --- | --- | --- | --- | --- | --- |
|  |  | **Reference = Male** | | | |
|  |  | Female | | Transgender/Non-binary/Other | |
|  |  | Estimate | P-value | Estimate | P-value |
| Adjusted 3-Month PROMIS Scores | | | | | |
| Higher Better | Cognitive Function | -4.12 | <0.001 | -8 | 0.017 |
|  | Physical Function | -2.07 | <0.001 | -4.73 | 0.04 |
|  | Social Participation | -2.82 | <0.001 | -8.6 | 0.004 |
| Lower Better | Anxiety | 2.82 | <0.001 | 6.49 | 0.023 |
|  | Depression | 1.71 | 0.006 | 7.65 | 0.004 |
|  | Fatigue | 5.05 | <0.001 | 12.75 | <0.001 |
|  | Sleep Disturbance | 1.68 | 0.004 | 4.11 | 0.097 |
|  | Pain Interference | 2.04 | 0.001 | 4.96 | 0.053 |
|  | Pain Intensity | 0.73 | <0.001 | 0.92 | 0.179 |
| Change in PROMIS scores additionally adjusted for baseline scores | |  |  |  |  |
| Higher Better | Cognitive Function | -1.43 | 0.023 | -3.73 | 0.163 |
|  | Physical Function | -1.13 | 0.023 | -3.11 | 0.142 |
|  | Social Participation | -1.63 | 0.011 | -7.63 | 0.005 |
| Lower Better | Anxiety | 0.95 | 0.091 | 2.82 | 0.24 |
|  | Depression | 0.74 | 0.136 | 4.19 | 0.051 |
|  | Fatigue | 3.15 | <0.001 | 8.24 | 0.002 |
|  | Sleep Disturbance | 0.94 | 0.051 | 2.57 | 0.214 |
|  | Pain Interference | 1.33 | 0.014 | 2.93 | 0.205 |
|  | Pain Intensity | 0.49 | 0.001 | 0.4 | 0.515 |

Note: The adjusted marginal differences of gender groups comparing with the non-Hispanic White participant group in COVID+ participants are calculated based on the adjusted estimates from the generalized linear models with adjustment for age, race/ethnicity, gender, education, marital status, health insurance status, family income, employment status, location of baseline testing, tobacco use, pre-existing health conditions, hospitalization, COVID vaccination status in addition to index COVID-19 test result and its interaction with gender group variables.

**Appendix 7.** Adjusted marginal differences in 3-month PROMIS domain scores and differences in change in scores from baseline to 3-month in race/ethnicity groups among COVID- participants reported with p-values

|  |  | **Estimates from Adjusted Marginal Difference with P-values** | | | | | | | |
| --- | --- | --- | --- | --- | --- | --- | --- | --- | --- |
|  |  | **Reference = Non-Hispanic White** | | | | | | | |
|  |  | Non-Hispanic Black | | Non-Hispanic Asian | | Hispanic/Latino | | Non-Hispanic Other | |
|  |  | Estimate | P-value | Estimate | P-value | Estimate | P-value | Estimate | P-value |
| Adjusted 3-Month PROMIS Scores | | | | | | | | | |
| Higher Better | Cognitive Function | 5.13 | 0.006 | 0.39 | 0.811 | 0.76 | 0.63 | -1.65 | 0.583 |
|  | Physical Function | 2.33 | 0.066 | 3.37 | 0.003 | -0.55 | 0.61 | 0.73 | 0.725 |
|  | Social Participation | 2.92 | 0.078 | 0.26 | 0.86 | -0.87 | 0.536 | -3.93 | 0.145 |
| Lower Better | Anxiety | -3.39 | 0.031 | -2.5 | 0.075 | -0.41 | 0.762 | 3.36 | 0.189 |
|  | Depression | -1.9 | 0.196 | -0.93 | 0.477 | -1.74 | 0.165 | 2.49 | 0.298 |
|  | Fatigue | -4.7 | 0.005 | -2.87 | 0.057 | -2.39 | 0.097 | 3.71 | 0.176 |
|  | Sleep Disturbance | -3.31 | 0.016 | 0.25 | 0.837 | -1.08 | 0.353 | -0.1 | 0.966 |
|  | Pain Interference | -0.55 | 0.7 | -1.91 | 0.13 | -0.3 | 0.802 | 2.4 | 0.298 |
|  | Pain Intensity | 0.56 | 0.14 | -0.5 | 0.141 | 0.01 | 0.976 | -0.19 | 0.764 |
| Change in PROMIS scores additionally adjusted for baseline scores | | | | | | | | | |
| Higher Better | Cognitive Function | 4.16 | 0.005 | 0.77 | 0.556 | -0.43 | 0.732 | 0.1 | 0.967 |
|  | Physical Function | 2.15 | 0.066 | 2.69 | 0.01 | -0.28 | 0.776 | 0.51 | 0.79 |
|  | Social Participation | 2.97 | 0.05 | 0.27 | 0.841 | -0.38 | 0.769 | -4.36 | 0.077 |
| Lower Better | Anxiety | -2.66 | 0.045 | -2.05 | 0.084 | 0.16 | 0.887 | 2.04 | 0.345 |
|  | Depression | -2.03 | 0.087 | -0.41 | 0.701 | 0.13 | 0.901 | 1.54 | 0.424 |
|  | Fatigue | -4.44 | 0.002 | -2.27 | 0.078 | -1.48 | 0.228 | 5.09 | 0.03 |
|  | Sleep Disturbance | -2.71 | 0.018 | 0.41 | 0.687 | -0.86 | 0.378 | 0.39 | 0.834 |
|  | Pain Interference | -0.43 | 0.736 | -1.08 | 0.345 | -0.61 | 0.576 | 2.97 | 0.153 |
|  | Pain Intensity | 0.32 | 0.336 | -0.4 | 0.189 | -0.25 | 0.388 | -0.03 | 0.96 |

Note: The adjusted marginal differences of racial-ethnic minoritized groups compared with the non-Hispanic White participant group in COVID- participants are calculated based on the adjusted estimates from the generalized linear models with adjustment for age, race/ethnicity, gender, education, marital status, health insurance status, family income, employment status, location of baseline testing, tobacco use, pre-existing health conditions, hospitalization, COVID vaccination status in addition to index COVID-19 test result and its interaction with race/ethnicity group variables.

**Appendix 8.** Adjusted marginal differences in 3-month PROMIS domain scores and differences in change in scores from baseline to 3-month in gender groups among COVID- participants reported with p-values

|  |  | **Estimates from Adjusted Marginal Difference with P-values** | | | |
| --- | --- | --- | --- | --- | --- |
|  |  | **Reference = Male** | | | |
|  |  | Female | | Transgender/Non-binary/Other | |
|  |  | Estimate | P-value | Estimate | P-value |
| Adjusted 3-Month PROMIS Scores | | | | | |
| Higher Better | Cognitive Function | -3.77 | 0.003 | -8.13 | 0.009 |
|  | Physical Function | -2.92 | 0.001 | -7.66 | <0.001 |
|  | Social Participation | -4.52 | <0.001 | -9.26 | 0.001 |
| Lower Better | Anxiety | 2.43 | 0.024 | 8.24 | 0.002 |
|  | Depression | 1.57 | 0.121 | 6.68 | 0.007 |
|  | Fatigue | 4.55 | <0.001 | 11.12 | <0.001 |
|  | Sleep Disturbance | 2.17 | 0.021 | 5.94 | 0.01 |
|  | Pain Interference | 2.32 | 0.017 | 6.65 | 0.005 |
|  | Pain Intensity | 0.71 | 0.007 | 1.29 | 0.045 |
| Change in PROMIS scores additionally adjusted for baseline scores | |  |  |  |  |
| Higher Better | Cognitive Function | -1.16 | 0.255 | -2.14 | 0.392 |
|  | Physical Function | -2.31 | 0.004 | -5.54 | 0.005 |
|  | Social Participation | -3.3 | 0.002 | -5.33 | 0.038 |
| Lower Better | Anxiety | -0.06 | 0.948 | 4.56 | 0.042 |
|  | Depression | -0.29 | 0.722 | 1.66 | 0.409 |
|  | Fatigue | 2.15 | 0.031 | 5.33 | 0.029 |
|  | Sleep Disturbance | 1.96 | 0.013 | 3.99 | 0.039 |
|  | Pain Interference | 1.52 | 0.085 | 5.08 | 0.019 |
|  | Pain Intensity | 0.51 | 0.029 | 1.1 | 0.054 |

Note: The adjusted marginal differences of gender groups compared with the non-Hispanic White participant group in COVID- participants are calculated based on the adjusted estimates from the generalized linear models with adjustment for age, race/ethnicity, gender, education, marital status, health insurance status, family income, employment status, location of baseline testing, tobacco use, pre-existing health conditions, hospitalization, COVID vaccination status in addition to index COVID-19 test result and its interaction with gender group variables.
