# Supplementary material for: Race, Ethnicity, and Gender Differences in Patient Reported Well-Being and Cognitive Functioning Within 3 Months of Symptomatic Illness During COVID-19 Pandemic
Source: J Racial Ethn Health Disparities. Author manuscript; Available in PMC 2025 Sep 19. (PMC11891493; doi:10.1007/s40615-024-02124-8)
Supplement: Supplementary file 1 [file NIHMS2057734-supplement-Supplements.docx]

**Appendix 1a.** Adjusted Marginal Difference in 3-month Score Estimation from the GLM

Example of calculating the marginal gender effects on 3-month score in each PROMIS outcomes: 

Using GLM, the regression on 3-month scores of each PROMIS domain or pain intensity is calculated as follows:

${(3-month score{Score}_{3m})}_{i}=β_{0 }+{female}_{i}*β_{1}+{other}_{i}*β_{2}+ {covid}_{i}*β_{3}+ {(female}_{i}*{covid}_{i}){*β}_{4}+ {(other}_{i}*{covid}_{i}){*β}_{5} + {{\boldsymbol{X}}_{\boldsymbol{i}}}^{\boldsymbol{'}}*\boldsymbol{B}$

Where ${\boldsymbol{X}}_{\boldsymbol{i}}$ denotes a vector of observed values of independent variables for patient,  $\boldsymbol{B}$  denotes these covariates’ coefficients for an outcome (e.g., physical function) and,

${covid}_{i}=\left\{\begin{aligned} 1,  &if COVID+ \\ 0,  &else \end{aligned}\right.$

${female}_{i}=\left\{\begin{aligned} 1,  &if gender is female \\ 0,  &else  \end{aligned}\right.$

${other}_{i}=\left\{\begin{aligned} 1,  &if gender is transgender/non-binary other  \\ 0,  &else  \end{aligned}\right.$

The estimated coefficients (hated-betas) from the GLM are used to calculate the adjusted marginal difference in 3-month score. For example, the adjusted marginal difference in 3-month score for physical function of female covid-positive participants in comparison to the 3-month score for physical function of male covid-positive participants is calculated as below:

${{3-month sScore_{3m}}_{physical\_function}(female vs. male)\vert}_{ COVID+}={\hat{β}}_{1}+ {\hat{β}}_{4}$ 

**Appendix 1b.** Adjusted Marginal Difference in Change in Score Estimation from the GLM

Example of calculating the marginal gender effects on change in each PROMIS outcomes from baseline to 3-month: 

Using GLM, the regression on change scores from baseline to 3 months of each PROMIS domain or pain intesity is calculated as follows:

${(change)}_{i}=β_{0 }+{female}_{i}*β_{1}+{other}_{i}*β_{2}+ {covid}_{i}*β_{3}+ {(female}_{i}*{covid}_{i}){*β}_{4}+ {(other}_{i}*{covid}_{i}){*β}_{5} + {BSScore}_{i}{*β}_{6}+ {{\boldsymbol{X}}_{\boldsymbol{i}}}^{\boldsymbol{'}}*\boldsymbol{B}$

Where ${\boldsymbol{BSScore}}_{\boldsymbol{i}}$ is the corresponding baseline PROMIS score, ${\boldsymbol{X}}_{\boldsymbol{i}}$ denotes a vector of observed values of independent variables for patient $i$, $\boldsymbol{B}$ denotes these covariates’ coefficients for outcome (e.g. physical function) and,

${covid}_{i}=\left\{\begin{aligned} 1,  &if COVID+ \\ 0,  &else \end{aligned}\right.$

${female}_{i}=\left\{\begin{aligned} 1,  &if gender is female \\ 0,  &else  \end{aligned}\right.$

${other}_{i}=\left\{\begin{aligned} 1,  &if gender is transgender/non-binary other  \\ 0,  &else  \end{aligned}\right.$

The estimated coefficients (hated-betas) from the GLM are used to calculate the adjusted marginal difference in change-score. For example, the adjusted marginal difference in change-score (from baseline to 3-months follow-up) for physical function of female covid-positive participants in comparison to the change-score for physical function of male covid-positive participants is calculated as below:

${{change}_{physical\_function}(female vs. male)\vert}_{ COVID+}={\hat{β}}_{1}+ {\hat{β}}_{4}$ 

**Appendix 2.** Adjusted marginal differences in 3-month PROMIS domain scores and differences in change in scores from baseline to 3-month in race/ethnicity groups among COVID- participants

|  |  | **Adjusted Marginal Difference (95% CI)**  **Reference = Non-Hispanic White** |  |  |  |
| --- | --- | --- | --- | --- | --- |
|  |  | Non-Hispanic Black | Non-Hispanic Asian | Hispanic/Latino | Non-Hispanic Other |
| Adjusted 3-Month PROMIS Scores |  |  |  |  |  |
| Higher Better | Cognitive Function | 5.13 (1.51, 8.75)^C^ | 0.39 (-2.84, 3.63) | 0.76 (-2.33, 3.85) | -1.65 (-7.53, 4.23) |
|  | Physical Function | 2.33 (-0.16, 4.83)^C^ | 3.37 (1.14, 5.59)^C^ | -0.55 (-2.68, 1.57) | 0.73 (-3.32, 4.77) |
|  | Social Participation | 2.92 (-0.33, 6.17)^C^ | 0.26 (-2.64, 3.16) | -0.87 (-3.65, 1.90) | -3.93 (-9.20, 1.35)^C^ |
| Lower Better | Anxiety | -3.39 (-6.48, -0.31)^C^ | -2.50 (-5.26, 0.25)^C^ | -0.41 (-3.04, 2.22) | 3.36 (-1.66, 8.37)^C^ |
|  | Depression | -1.90 (-4.78, 0.98) | -0.93 (-3.51, 1.64) | -1.74 (-4.20, 0.72) | 2.49 (-2.20, 7.17)^C^ |
|  | Fatigue | -4.70 (-8.01, -1.39)^C^ | -2.87 (-5.82, 0.09)^C^ | -2.39 (-5.22, 0.44)^C^ | 3.71 (-1.67, 9.09)^C^ |
|  | Sleep Disturbance | -3.31 (-6.00, -0.63)^C^ | 0.25 (-2.14, 2.65) | -1.08 (-3.37, 1.20) | -0.10 (-4.46, 4.26) |
|  | Pain Interference | -0.55 (-3.33, 2.23) | -1.91 (-4.40, 0.57) | -0.30 (-2.67, 2.07) | 2.40 (-2.12, 6.91)^C^ |
|  | Pain Intensity | 0.56 (-0.18, 1.31) | -0.50 (-1.17, 0.17) | 0.01 (-0.63, 0.65) | -0.19 (-1.40, 1.03) |
| Change in PROMIS scores additionally adjusted for baseline scores |  |  |  |  |  |
| Higher Better | Cognitive Function | 4.16 (1.27, 7.05)^C^ | 0.77 (-1.81, 3.36) | -0.43 (-2.90, 2.04) | 0.10 (-4.60, 4.80) |
|  | Physical Function | 2.15 (-0.14, 4.45)^C^ | 2.69 (0.63, 4.74)^C^ | -0.28 (-2.24, 1.68) | 0.51 (-3.22, 4.24) |
|  | Social Participation | 2.97 (-0.00, 5.95)^C^ | 0.27 (-2.39, 2.93) | -0.38 (-2.92, 2.16) | -4.36 (-9.19, 0.47)^C^ |
| Lower Better | Anxiety | -2.66 (-5.26, -0.06)^C^ | -2.05 (-4.37, 0.27)^C^ | 0.16 (-2.06, 2.38) | 2.04 (-2.19, 6.26)^C^ |
|  | Depression | -2.03 (-4.36, 0.30)^C^ | -0.41 (-2.49, 1.67) | 0.13 (-1.87, 2.12) | 1.54 (-2.24, 5.33) |
|  | Fatigue | -4.44 (-7.27, -1.62)^C^ | -2.27 (-4.79, 0.25)^C^ | -1.48 (-3.89, 0.93) | 5.09 (0.50, 9.68)^C^ |
|  | Sleep Disturbance | -2.71 (-4.95, -0.47)^C^ | 0.41 (-1.59, 2.41) | -0.86 (-2.77, 1.05) | 0.39 (-3.25, 4.02) |
|  | Pain Interference | -0.43 (-2.94, 2.08) | -1.08 (-3.32, 1.16) | -0.61 (-2.75, 1.53) | 2.97 (-1.11, 7.05)^C^ |
|  | Pain Intensity | 0.32 (-0.34, 0.98) | -0.40 (-0.99, 0.20) | -0.25 (-0.81, 0.32) | -0.03 (-1.10, 1.04) |

Note: (a) The adjusted marginal differences of racial-ethnic minoritized groups compared with the non-Hispanic White participant group in COVID- participants are calculated based on the adjusted estimates from the generalized linear models with adjustment for age, race/ethnicity, gender, education, marital status, health insurance status, family income, employment status, location of baseline testing, tobacco use, pre-existing health conditions, hospitalization, COVID vaccination status in addition to index COVID-19 test result and its interaction with race/ethnicity group variables.

(b)Clinical significance indicated by ‘C’ in the superscript. For scores other than pain intensity, a difference in score of ≥2 is considered clinically significant and for pain intensity, a score difference of ≥ 1 is considered clinically significant.

**Appendix 3**. Adjusted marginal differences in 3-month PROMIS domain scores and differences in change in scores from baseline to 3-month in gender groups among COVID- participants

|  |  | **Adjusted Marginal Difference (95% CI)**  **Reference = Male** |  |
| --- | --- | --- | --- |
|  |  | Female | Transgender/Non-binary/Other |
| 3-Month PROMIS Scores |  |  |  |
| Higher Better | Cognitive Function | -3.77 (-6.26, -1.29)^C^ | -8.13 (-14.24, -2.02)^C^ |
|  | Physical Function | -2.92 (-4.63, -1.20)^C^ | -7.66 (-11.87, -3.44)^C^ |
|  | Social Participation | -4.52 (-6.75, -2.29)^C^ | -9.26 (-14.74, -3.79)^C^ |
| Lower Better | Anxiety | 2.43 (0.31, 4.55)^C^ | 8.24 (3.04, 13.45)^C^ |
|  | Depression | 1.57 (-0.41, 3.54) | 6.68 (1.81, 11.55)^C^ |
|  | Fatigue | 4.55 (2.28, 6.82)^C^ | 11.12 (5.53, 16.71)^C^ |
|  | Sleep Disturbance | 2.17 (0.32, 4.01)^C^ | 5.94 (1.41, 10.48)^C^ |
|  | Pain Interference | 2.32 (0.42, 4.23)^C^ | 6.65 (1.96, 11.34)^C^ |
|  | Pain Intensity | 0.71 (0.20, 1.22) | 1.29 (0.03, 2.55)^C^ |
| Change in PROMIS scores additionally adjusted for baseline scores |  |  |  |
| Higher Better | Cognitive Function | -1.16 (-3.16, 0.84) | -2.14 (-7.05, 2.77)^C^ |
|  | Physical Function | -2.31 (-3.90, -0.73)^C^ | -5.54 (-9.43, -1.65)^C^ |
|  | Social Participation | -3.30 (-5.35, -1.26)^C^ | -5.33 (-10.38, -0.29)^C^ |
| Lower Better | Anxiety | -0.06 (-1.86, 1.74) | 4.56 (0.16, 8.96)^C^ |
|  | Depression | -0.29 (-1.89, 1.31) | 1.66 (-2.29, 5.61) |
|  | Fatigue | 2.15 (0.20, 4.10)^C^ | 5.33 (0.53, 10.12)^C^ |
|  | Sleep Disturbance | 1.96 (0.42, 3.50) | 3.99 (0.20, 7.77)^C^ |
|  | Pain Interference | 1.52 (-0.21, 3.24) | 5.08 (0.84, 9.32)^C^ |
|  | Pain Intensity | 0.51 (0.05, 0.96) | 1.10 (-0.02, 2.21)^C^ |

Note: (a) The adjusted marginal differences of gender groups compared with the non-Hispanic White participant group in COVID- participants are calculated based on the adjusted estimates from the generalized linear models with adjustment for age, race/ethnicity, gender, education, marital status, health insurance status, family income, employment status, location of baseline testing, tobacco use, pre-existing health conditions, hospitalization, COVID vaccination status in addition to index COVID-19 test result and its interaction with gender group variables.

(b)Clinical significance indicated by ‘C’ in the superscript. For scores other than pain intensity, a difference in score of ≥2 is considered clinically significant and for pain intensity, a score difference of ≥ 1 is considered clinically significant.
